# Synthesis and Magnetic Properties of the Multiferroic [C(NH_2_)_3_]Cr(HCOO)_3_ Metal–Organic Framework: The Role of Spin–Orbit Coupling and Jahn–Teller Distortions

**DOI:** 10.1021/acs.inorgchem.3c02557

**Published:** 2023-10-11

**Authors:** Kunihiro Yananose, Ewan R. Clark, Paul J. Saines, Paolo Barone, Alessandro Stroppa, Jaejun Yu

**Affiliations:** † Korea Institute for Advanced Study, Seoul 02455, Republic of Korea; ‡ Center for Theoretical Physics, Department of Physics and Astronomy, Seoul National University, Seoul 08826, Republic of Korea; § School of Chemistry and Forensic Science, University of Kent, Canterbury CT2 7NH, U.K.; ∥ Consiglio Nazionale delle Ricerche, Institute for Superconducting and Innovative Materials and Devices (CNR-SPIN), Area della Ricerca di Tor Vergata, Via del Fosso del Cavaliere 100, 00133 Rome, Italy; ⊥ Consiglio Nazionale delle Ricerche, Institute for Superconducting and Innovative Materials and Devices (CNR-SPIN) c/o Department of Physical and Chemical Sciences, University of L’Aquila, Via Vetoio, I-67100 Coppito, L’Aquila, Italy

## Abstract

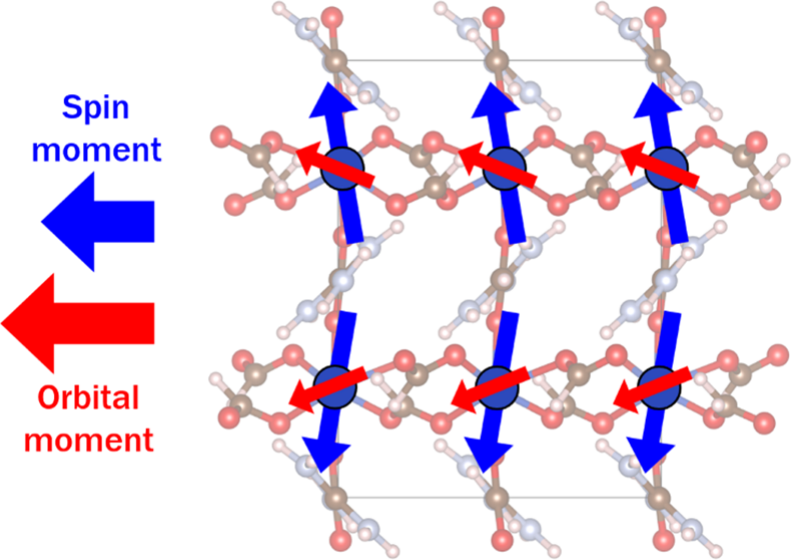

We report for the first time the synthesis of [C(NH_2_)_3_]Cr(HCOO)_3_ stabilizing Cr^2+^ in formate perovskite, which adopts a polar structure and orders magnetically below 8 K. We discuss in detail the magnetic properties and their coupling to the crystal structure based on first-principles calculations, symmetry, and model Hamiltonian analysis. We establish a general model for the orbital magnetic moment of [C(NH_2_)_3_]M(HCOO)_3_ (M = Cr, Cu) based on perturbation theory, revealing the key role of the Jahn–Teller distortions. We also analyze their spin and orbital textures in *k*-space, which show unique characteristics.

## Abstract 2

Among the multiferroic metal−organic framework [C(NH_2_)_3_]M(HCOO)_3_ family, where M is a 3d transition metal, the M = Cr case was missing for more than 10 years, although its existence and properties were predicted in 2013. We report the synthesis, structure, and magnetic properties of the M = Cr case, [C(NH_2_)_3_]Cr(HCOO)_3_. Density functional theory calculations and model studies support our observation. In addition, the M = Cu case is suggested to exhibit orbital-dominant weak ferromagnetism.

## Introduction

Metal–organic frameworks (MOFs) are materials in which metal ions are connected with each other by organic molecules. The choice of organic linkers allows for a great variety in their crystal structures. One of its classes, the porous MOFs hold a large portion of cavities in them. Their tunable porosity enables applications in gas storage, catalysis, etc. Thus, they are widely studied.^1,2^ On the other hand, dense MOFs feature much smaller cavities in comparison to porous MOFs; their cations are closer together and can thus play a significant role in the emergence of their functional properties.^3−5^ The combination of organic–inorganic features can induce both magnetism and ferroelectricity simultaneously, i.e., multiferroicity. In some multiferroic materials, the ferroelectric and magnetic orders are coupled, giving rise to the possibility of controlling the magnetic property by the electric field and vice versa.^6−8^ Thus, both the magnetic and electric orders, their coupling, and the role and control of the structural deformation are important aspects for dense MOFs.^9−24^

**Figure 1:**
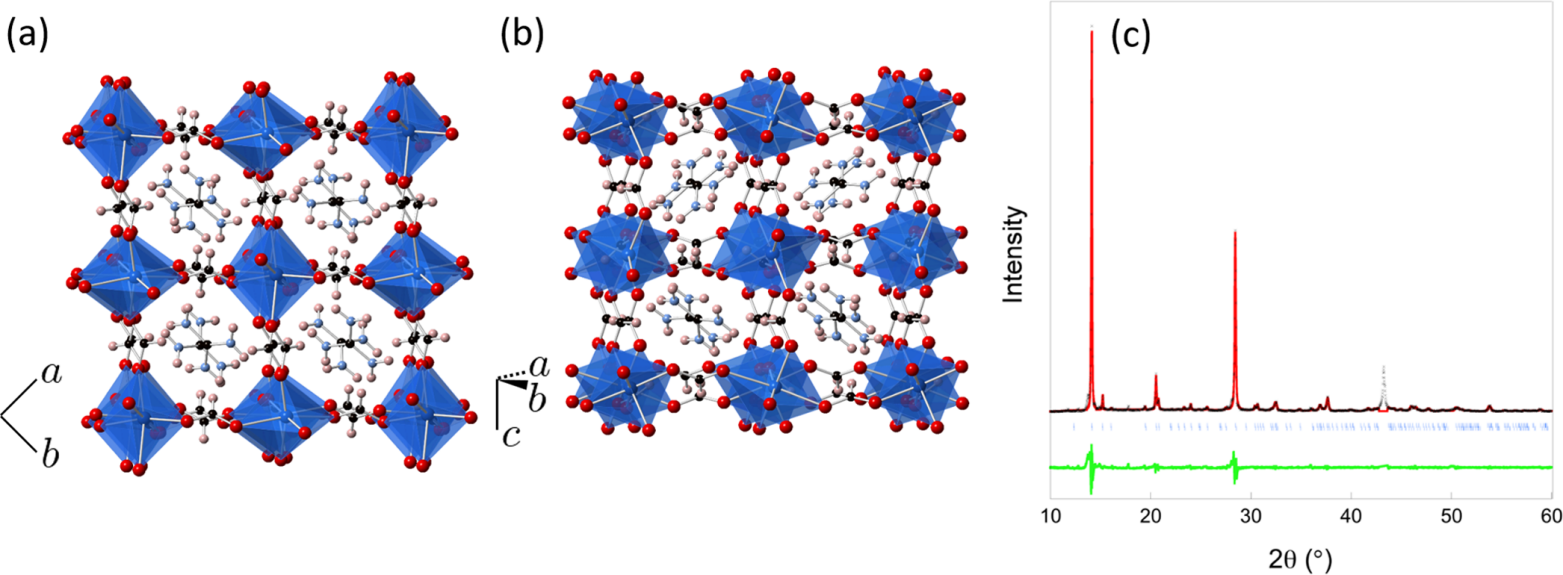
(a, b) Crystal structure of **1-Cr** at 300 K seen from different directions indicated by the crystal axes in each panel with the Cr octahedra shown in blue and carbon, hydrogen, nitrogen, and oxygen atoms shown as black, pink, light blue, and red spheres, respectively. (c) Le Bail fit to a powder X-ray diffraction pattern of **1-Cr** with the experimental data shown as black crosses, the calculated and difference intensities shown as red and green lines, respectively, and the vertical blue markers noting the expected position of Bragg reflections. The omitted peak from the fit was confirmed as being associated with the air-sensitive sample holder, likely from a Ni-based alloy. *R*_p_, *R*_wp_, and χ^2^ of the fit were 5.89, 9.81, and 8.29, respectively.

Among dense MOFs, the [C(NH_2_)_3_]M(HCOO)_3_ (M = Mn, Fe, Co, Ni, Cu, and Zn) series have been synthesized with a perovskite-type ABX_3_ structure.^25^ They have the guanidinium (Gua) ion (C(NH_2_)_3_)^+^ on the A site, the 3d transition metal ions (M^2+^) occupying the B site, and formate HCOO^–^ ions as the X site anions, as shown in Figure 1(a,b). These materials show magnetic ordering of their M^2+^ ions; notably, only the Cu-based analogue (hereafter denoted as **1-Cu**), featuring the Jahn–Teller (JT) active ion Cu^2+^ (*d*^9^), displays a polar structure and a weak ferromagnetic component that has been proposed to correlate with JT distortions.^9,25^ Similarly, a theoretical study on the not yet synthesized perovskite MOF with M = Cr^2+^ (hereafter denoted as **1-Cr**) proposed a nontrivial role of the Cr^2+^ (*d*^4^) JT active ion in shaping both ferroelectric and (weak) ferromagnetic properties.^12^ On one hand, the nonpolar JT distortions couple to another nonpolar mode giving rise to a polar hybrid mode which breaks inversion symmetry, resulting in a so-called hybrid improper ferroelectricity.^26−29^ On the other hand, the weak ferromagnetic component was proposed to arise from the JT-related orbital ordering and its interplay with spin–orbit coupling (SOC).^12^ As opposed to most of their inorganic counterparts, the multiferroic phase of ABX_3_**1-Cu** and **1-Cr** holds the promise of a stronger coupling between magnetic and ferroelectric properties, as both functional properties share the JT activity as a common origin.^9,12,14^ Although **1-Cr** was theoretically predicted to display multiferroic properties like the similar **1-Cu**, it was not previously possible to synthesize it due to the significant difficulties encountered in stabilizing Cr^2+^, which undergoes rapid oxidation in air to the more stable trivalent oxidation state. Furthermore, recent experimental studies of multiferroic Cr^2+^ halides with layers^30^ or chains^31^ of Cr octahedra further emphasize the potential for unexploited functionality in Cr^2+^ hybrid perovskites.

In this study, we successfully synthesized **1-Cr** for the first time, confirmed its polar structure, and measured its magnetic properties. The structural analysis confirms the theoretical predictions, while we observe an antiferromagnetic transition at *T*_c_ ∼ 8 K, roughly twice the Néel temperature of **1-Cu** but four times smaller than the theoretical prediction, with no evident signatures of weak ferromagnetism (WFM). We accordingly revised the earlier magnetic model^12^ by proposing a new and more accurate set of magnetic parameters extracted from first-principles calculations, which are able to reproduce the experimental measurements as well as the magnetic transition temperature more accurately. Noticeably, the revised model, which is based on the subtle interplay of orbital order, SOC, and structural distortions, is able to capture the complex magnetic configuration of **1-Cr**. The key role of SOC, so commonly neglected in 3d transition metals, in the magnetic properties of **1-Cr** and **1-Cu**, is emphasized by the significant contribution this makes to their effective magnetic moments; particularly in **1-Cu**, where the orbital ferromagnetic component is even comparable to the spin contribution. We explain the orbital magnetic moment, previously neglected in **1-Cr/Cu**, and its dependence on orbital ordering based on the second-order perturbation theory and the JT effective Hamiltonian. Our microscopic model is in very good agreement with the density functional theory (DFT) calculation results and could be applied to similar compounds. Finally, we also analyze the spin and orbital textures in *k*-space, which show unique characteristics.

## Methods

All synthetic procedures were performed according to standard Schlenk line procedures under an atmosphere of dry argon. Isolated Cr^2+^ samples were stored in an argon glovebox, and samples for powder X-ray diffraction and SQUID magnetometry analysis were placed in the same glovebox. This was essential to prevent the oxidation of Cr^2+^ to a more stable trivalent oxidation state. **Caution!***Chromium salts are known sensitizers, and so care must be taken to avoid generating any loose dust and to dispose of all samples appropriately. Multiple reactions described herein involve the release of a condensable gas while working on a Schlenk line; care should be taken both to prevent sealing reaction vessels to avoid pressure buildup and to ensure that cryogenic traps can vent safely on warming.*

Cyan blocklike crystals of **1-Cr** suitable for structural determination were made by layering a colorless solution of [C(NH_2_)_3_](HCOO) (0.420 g, 4 mmol) (see Section S1 of the Supporting Information (SI) for the preparation method) and H_2_COO (165 μL, 4 mmol) in 50:50 v/v water/ethanol upon a pale blue solution of CrCl_2_ (61 mg, 0.5 mmol) in water (2 cm^3^). This produced an intense purple interface with crystals formed after standing for a week. A bulk sample suitable for further analysis was made by the addition of an aqueous solution of CrSO_4_·5H_2_O, made using literature methods^32^ (0.5 M, 5 cm^3^), into an aqueous solution of [C(NH_2_)_3_](HCOO) (see Section S1 for further details) in a single portion. This was stirred briefly to give a homogeneous purple solution, which, on standing overnight, transformed to a pale blue supernatant above a cyan solid. The solid was isolated by filtration, washed with EtOH (2 × 10 cm^3^), and dried in vacuo to give **1-Cr** as a cyan microcrystalline powder (0.541 mg, 2.1 mmol, 84% yield.)

Structure determination was carried out using single crystal X-ray diffraction (SCXRD) data recorded on an Agilent SuperNova Dual Source diffractometer (see Section S2 for further details of experimental and structure solution methods). Structures from this experiment are deposited in the CSD as entries 2278325–2278331. Bulk purity of the sample was assessed using powder X-ray diffraction (PXRD) collected using a Rigaku Miniflex using Cu Kα (40 kV, 15 mA) with the sample mounted in a propriety air-sensitive sample holder. A Le Bail fit was carried out using the program Rietica.^33^ Elemental microanalysis was carried out at the London Metropolitan University. Magnetometric studies were performed using a Quantum Design MPMS 7 magnetometer, utilizing an applied field of 1000 Oe for variable temperature susceptibility (χ) measurements. Samples were placed in gelatin capsules enclosed inside a pierced straw tube with a uniform diamagnetic background. Differential scanning calorimetry (DSC) measurements were performed on **1-Cr** using a NETZSCH DSC 200PC with the sample in a closed Al pan. Sample loading and data collection were performed under an inert nitrogen atmosphere.

We used the Vienna Ab Initio Simulation Package (VASP)^34−37^ for the first-principles DFT calculation. To include SOC, we performed noncollinear spin DFT calculations. Generalized gradient approximation by Perdew–Burke–Ernzerhof (GGA-PBE) for the exchange–correlation functional^38^ and the projector augmented wave pseudopotential^39^ were adopted. The plane wave energy cutoff was chosen to be 500 eV. A 4 × 4 × 4 regular *k*-space grid was used. For the lattice constants, experimental values of **1-Cu***a* = 8.5212 Å, *b* = 9.0321 Å, and *c* = 11.3497 Å from ref (25) were used for both **1-Cr** and **1-Cu** for consistency with the previous theoretical works.^9,12^ Note that the measured lattice constants of **1-Cr** are close to these values, thus justifying our initial guess (see Section S2). For a ferroelectric structure, one can define a corresponding paraelectric virtual structure of higher symmetry, referred to as the pseudosymmetric structure, using the group-theoretical method implemented in the software PSEUDO of the Bilbao Crystallography server.^40^

We estimated the transition temperature (*T*_c_) by using the spin model suggested in ref (12) and the Monte Carlo simulations adopting a standard Metropolis algorithm. We additionally considered the on-site Coulomb energy correction in DFT (DFT + *U* + *J* calculations^41^), which was neglected in previous calculations,^12^ when obtaining the spin model parameters. Two sets of parameters (*U*, *J*) = (2.5, 0.5) and (3.0, 1.0) (eV) were used. Further details of the spin model can be found in Sections S4 and S5 of the SI.

## Results and Discussion

### Synthesis and Crystal Structure

Initial syntheses of **1-Cr** followed a modified preparation from those used for the Co and Fe analogues,^25^ but yields were low, and sample purity was poor as a result of the contamination from Cr^3+^ within the commercial source. Nevertheless, slow diffusion of a solution of [C(NH_2_)_3_](HCOO) in ethanol into a solution of chromous/chromic chloride in water allowed the growth of single crystals of sufficient quality for SCXRD studies. Attempts to scale this method up produced inseparable mixtures of crystals, attributed to the Cr^3+^ impurities in the starting material, as suggested by the pale color of the Cr solution since fresh Cr^2+^Cl_2_ solutions are an intense royal blue. To avoid this problem, efforts to make bulk samples of **1-Cr** for further analysis utilized CrSO_4_·5H_2_O in an aqueous medium. Fits to PXRD patterns indicated that this method resulted in a sample with a structure consistent with that determined from SCXRD with only trace amounts of an unidentified impurity phase (see Figure 1(c)). Lattice parameters were determined to be *a* = 8.6358(8) Å, *b* = 11.6480(15) Å, and *c* = 9.1050(9) Å, yielding a unit cell volume of 915.87(15) Å^3^. The purity of this sample was further confirmed by elemental analysis results (experimental values of C 19.47, H 3.57, and N 17.54% to calculated values of 19.44, 3.67, and 17.00%, respectively).

Examination of the systematic absences of the SCXRD data of **1-Cr** collected at 100 K indicates that it adopts a structure in either the *Pna*2_1_ or *Pnma* orthorhombic symmetry, with the systematic absences required for the *Pnan* structure adopted by the [C(NH_2_)_3_]M(HCOO)_3_ (M = Mn–Ni and Zn) compounds violated.^25^ Extensive attempts to solve the structure were successful only for *Pna*2_1_ symmetry, with *Pnma* not giving a chemically sensible or even a complete solution. This is consistent with the previous report of **1-Cu** adopting *Pna*2_1_ symmetry and previous combined first-principles calculations and group-theoretical analysis of **1-Cr** indicating this to be the expected symmetry of this material.^9,12,25^ We should note that the crystal used in this study was a twin by inversion, which may complicate using them to experimentally confirm ferroelectric switching.

As expected, **1-Cr** adopts a hybrid perovskite structure which closely resembles other [C(NH_2_)_3_]M(HCOO)_3_ phases, including a similar conventional *a*^–^*a*^–^*c*^–^ tilt system^25^ (see Figure 1 for the crystal structure and Figure S1 in the SI for the asymmetric unit). As previously seen in isostructural **1-Cu** where the *d*^9^ Cu^2+^ cations also have a JT-distorted bonding environment (see Table S2 for bond angles), the elongated axis of the *d*^4^ Cr^2+^O_6_ octahedra also alternates within the *ab*-plane. This is in a Cr–O_short_···Cr–O_long_···Cr–O_short_···Cr–O_long_ pattern between neighboring octahedra connected by formates along both orthogonal directions in this plane; at 300 K, the Cr–O_short_ distances in this plane are 2.031(9) and 2.066(8) Å, while the Cr–O_long_ distances are 2.347(9) and 2.390(8) Å (see Figure S2 for evolution with temperature). The remaining two Cr–O_short_ bond distances required to complete a conventional *trans*-elongated JT-distorted octahedra are oriented along the *c*-axis with distances of 2.065(14) and 2.079(15) Å. The bond valence sum of the Cr cation is 2.00, consistent with the expected divalent oxidation state.^42^ Along with the JT distortion of the octahedra in **1-Cr**, the main difference in the structure of this phase when compared to the *Pnan* [C(NH_2_)_3_]M(HCOO)_3_ frameworks is the subtle rotations of the Gua cations along the *a-* and *c*-axis.^12,25^

As is observed for other [C(NH_2_)_3_]M(HCOO)_3_ phases, variable temperature SCXRD analysis indicates that Gua remains ordered up to 400 K, the highest temperature measured during this study.^25^ At 400 K, a significant decrease in the data quality of variable temperature SCXRD is noted (the average *I*/σ declines from being consistently >15.9 to 11.9 at 400 K), which may indicate that the measurements are approaching a temperature at which the material begins to lose crystallinity. At this temperature, the reflections indicating the violation of the systematic absences associated with the second *n*-glide required for *Pnan* symmetry are lost. While this is most likely a result of the poorer data quality available from this air-sensitive sample above ambient temperature, there is a significant decrease in the JT distortion at the same temperature (see Figure S2), which could alternatively indicate the onset of a transition to *Pnan* symmetry.

**Figure 2:**
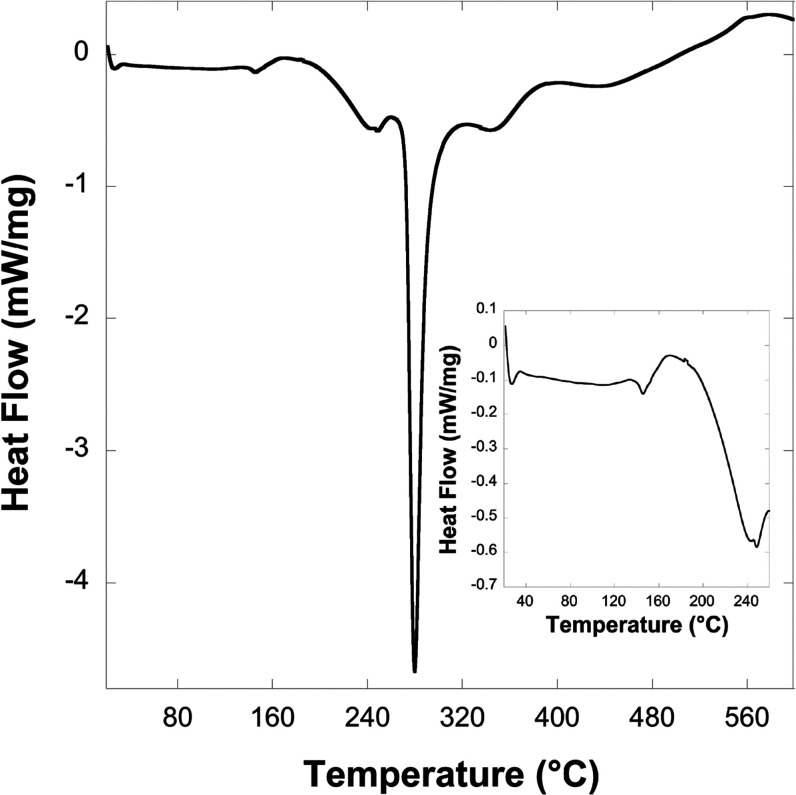
Differential scanning calorimetry trace of **1-Cr** measured up to 600 °C. The inset highlights the signal at a lower temperature.

DSC analysis of **1-Cr** shown in Figure 2 indicates a possible phase transition near 140 °C with an enthalpy change of approximately −0.9 kJ mol^–1^. This may suggest that the weakening in the JT distortion of **1-Cr** identified in the crystal structure at 400 K may be a precursor to a phase transition related to the JT distortion. The enthalpy change measured here is at least an order of magnitude lower than the JT energies of [Cr(H_2_O)_6_]^2+^ suggested by calculations and spectroscopic measurements.^43,44^ This suggests that if this transition is related to an apparent loss of the JT distortion in the crystal structure, it is likely related to a loss of orbital order rather than quenching of the JT effect, i.e., a loss of ordering between the JT-distorted local structures. There are three further peaks indicative of endothermic processes in **1-Cr** at higher temperatures, centered near 249, 272, and 335 °C with estimated enthalpy changes of −7.0, −65.2, and −4.3 kJ mol^–1^, respectively. The temperatures at which these features appear are broadly consistent with the reported decomposition temperatures of the Mn, Fe, and Co analogues, so we anticipate these features may also indicate the decomposition of **1-Cr**.^25^

The thermal expansion of this material across the 100–400 K range studied in this study is anisotropic with a significant positive thermal expansion of 50(5) and 29(7) M K^–1^ observed along the *a*- and *c*-axis, while a modest negative thermal expansion of −14(3) M K^–1^ is measured along the *b*-axis (see Figure S3 for the lattice parameter plot). This is consistent with the orientation of analogous anisotropic thermal expansion observed from other members of the [C(NH_2_)_3_]M(HCOO)_3_ series,^45^ appearing to be of a similar scale to the Mn analogue which has the highest anisotropic negative thermal expansion of the members reported experimentally thus far and related [C(NH_2_)_3_]Er(HCOO)_2_(C_2_O_4_).^46^ Framework hinging combines with the expansion of the M-formate-M struts upon heating to govern the overall observed thermal expansivities. For other members of the [C(NH_2_)_3_]M(HCOO)_3_ series, the anisotropic thermal expansion has been attributed to hinging of the metal-formate framework as the pore shape becomes more isotropic on heating.^45,46^ In the case of **1-Cr**, this leads to a greater expansion of the *a*-axis, along which the hinging angles are acute, accompanied by a more modest contraction along the *b*-axis, along which the hinging angles are obtuse (see Figure S4). The connectivity of the octahedra along the *c*-axis by the formate ligands means that expansion in this direction is only controlled by the strut expansion as the framework cannot hinge in this direction, unlike the *ab*-plane where the connectivity via the formate ligands is approximately along the ⟨110⟩ directions.

### Magnetic Properties

**Figure 3:**
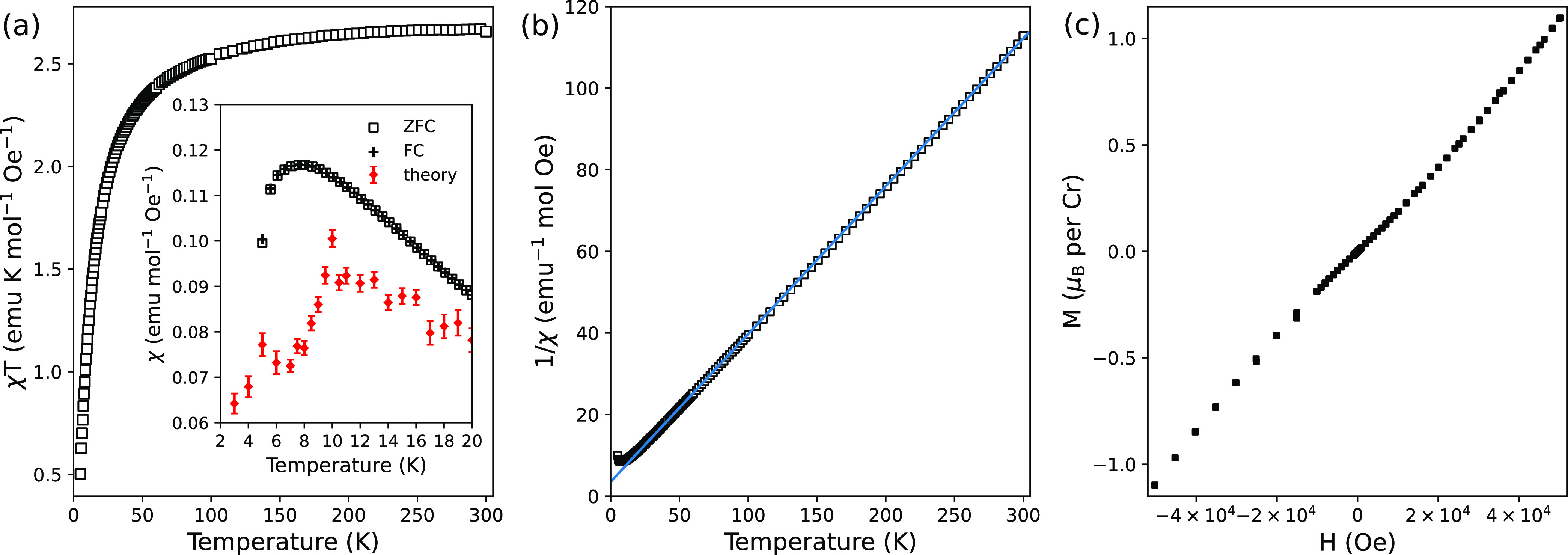
(a) Plot of χ*T* versus temperature for **1-Cr** in a 1000 Oe field. The inset shows the ZFC and FC χ measurements measured between 5 and 20 K and the predicted χ by the MC simulation. (b) Plot of 1/χ versus temperature for **1-Cr**, which is well fitted by a linear trend line (shown in blue) from 20 to 300 K. (c) Plot of magnetization, M, of **1-Cr** versus the applied field, H, at 5 K.

Field cooled (FC) and zero field cooled (ZFC) χ measurements with respect to temperature in a 1000 Oe applied magnetic field indicate maxima just below 8 K, with no divergence of these measurements below this temperature (see Figure 3(a)). This indicates a transition to a compensated antiferromagnetic (AFM) state. A plot of 1/χ with temperature is well fitted by a linear trend between 20 and 300 K with deviations observed below 20 K, consistent with AFM correlations (see Figure 3(b)). This fit yields a Weiss constant, Θ, of −9.8 K, consistent with the observed AFM transition being slightly below this. This fit also indicated an effective magnetic moment of 4.70 μ_B_, modestly below the spin-only magnetic moment expected for a high spin *d*^4^ cation, 4.90 μ_B_, but consistent with the reduced value observed for other scarce examples of octahedral Cr^2+^ compounds with group 16-based ligands, including Cr_3_(PO_4_)_2_ (4.50 μ_B_)^47^ and Y_2_CrS_4_ (4.70 μ_B_).^48^ χ*T* gradually declines from a value consistent with noninteracting Cr^2+^ cations at 300 to 90 K before decreasing more rapidly to 5 K, consistent with the strong AFM coupling emerging at low temperatures (see Figure 3(a)). Consistent with an AFM state, isothermal magnetization measurements at 5 K gradually increase as a function of the applied magnetic field, reaching a value of 1.10 μ_B_ per Cr^2+^ cation at 50 kOe, with no indication of hysteresis observed (see Figure 3(c)). Determining the magnetic structure of **1-Cr** using neutron diffraction would be desirable in future work in order to gain more insights into the magnetic structure at very low temperatures. This is not a trivial task due to the need to make more than a gram of a perdeuterated sample of this air-sensitive material.

### First-Principles Calculations and Model Study of Magnetic Properties

#### Definition of Structural Interpolation

**Figure 4:**
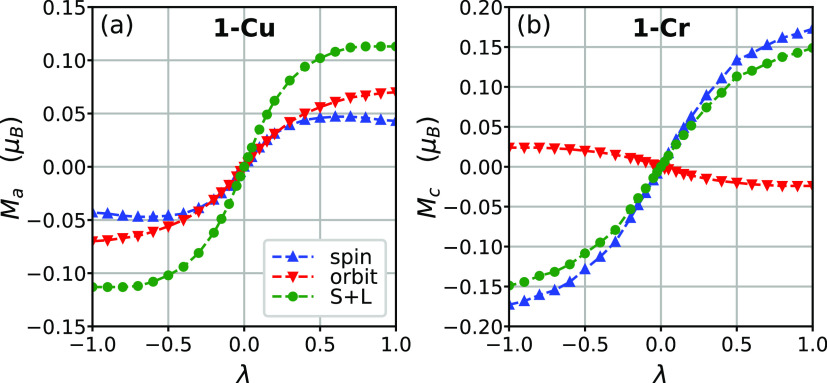
Spin, orbital, and total (S + L) magnetic moment per unit cell of (a) **1-Cu** and (b) **1-Cr** with respect to the structure parameter λ. For **1-Cu** (**1-Cr**), the *a*(*c*)-component is shown.

The space group symmetry of JT-distorted **1-Cr/Cu** is the polar *Pna*2_1_ (No. 33) with the corresponding centrosymmetric pseudosymmetric group *Pnan* (No. 52, *Pnna* in standard settings). The structural distortion relating the *Pnan* to *Pna*2_1_ structure can be expressed using the interpolation parameter λ^9,12^ (λ = 0 for *Pnan* and λ = 1 for the original *Pna*2_1_ structure). We denote the atomic positions at λ = 0 as **r**_*Pnan*_ and the displacement vectors from the λ = 0 structure to the λ = 1 structure as **u**. Then, the atomic positions of the interpolated structure are written as **r**(λ) = **r**_*Pnan*_ + λ**u**. The structural path defined by λ evolving from +1 to −1 represents a polarization switching path, passing through the centrosymmetric *Pnan* (λ = 0). Moreover, the net magnetic moment also reverses its direction along the path,^9,12^ as reproduced in Figure 4. Notably, for **1-Cr**, the predicted structure^12^ is in good agreement with the new experimental structure in terms of the space group symmetry, JT distortions, and structural details (see Section S2).

#### Magnetic Properties

As the unit cell of *Pna*2_1_**1-Cr/Cu** comprises four magnetic ions, we can define four magnetic order parameters per cell compatible with nonmagnetic translational symmetries (see Section S3), describing a ferromagnetic configuration ***M*** and three antiferromagnetic configurations ***G***, ***A***, and ***C***. While all magnetic moments are antiparallel in the G-type AFM configuration, the A-type AFM (A-AFM) structure is characterized by layers of parallel magnetic moments antiferromagnetically aligned, and the C-type comprises ferromagnetic chains that are antiferromagnetically coupled (see Figure S5).

In **1-Cr/Cu**, the antiferro-distortive ordering of the octahedra induces an orbital ordering; i.e., the cooperative JT effect determines the orbital structure characterized by intralayer antiferro-orbital ordering and interlayer ferro-orbital ordering. Accordingly, the Goodenough–Kanamori–Anderson rule^49,50^ predicts the FM interaction between the in-plane neighboring ions and the AFM interaction between the out-of-plane neighboring ions, i.e., A-AFM. Our new DFT (+*U* + *J*) calculations indeed confirm this scenario, in agreement with previous studies.^9,12^ Noticeably, a weak ferromagnetic component, appearing as a secondary order parameter, is allowed only for *Pn*′*a*′2_1_: {*A*_*a*_; *G*_*b*_, *M*_*c*_} and *Pna*′2_1_′: {*A*_*c*_; *C*_*b*_, *M*_*a*_}, where the prime means that the symmetry operation is accompanied by the time-reversal operation. The spin axes of the AFM alignment identified by the new calculations are the crystallographic *c*-axis for **1-Cu** (*Pna*′2_1_′) and *a*-axis for **1-Cr** (*Pn*′*a*′2_1_), consistent with previous studies.^9,12^ However, there also exist secondary AFM orders *G*_*b*_ and *C*_*b*_, which were previously overlooked.^9,12^ A weak ferromagnetic moment is then allowed by the magnetic space symmetries to develop along the *a*-axis in **1-Cu** and along the *c*-axis in **1-Cr**, which can be viewed as resulting from a small canting of the primary A-AFM configuration. The experiments observed the WFM moment of **1-Cu**,^25,51^ while no evident signature for WFM appears from our new measurements for **1-Cr**. This discrepancy motivated us to revisit the magnetic model proposed earlier by using more accurate parameters, as derived from the present work.

The spin canting can be attributed to the magnetic single-ion anisotropy (MSIA) originating from SOC,^12^ ruling out the antisymmetric Dzyaloshinskii–Moriya exchange interaction^63,64^ since it is incompatible with the {*A*_*a*_, *M*_*c*_} and {*A*_*c*_, *M*_*a*_} coupling (see Sections S4 and S10). The antiferro-orbital ordering within the *ab*-plane is responsible for different anisotropy axes on neighboring magnetic sites, which may cause a canting of the primary magnetic configuration.^12,52^ The rotation of the orbital order/JT distortion pattern, accompanying the switching of λ from 1 to −1, which was shown in ref (12), can then be naturally related to a rotation of the local anisotropy axes and a corresponding switching of the magnetic moment, as shown by the DFT calculations (Figure 4).

The calculated WFM spin magnetic moments are shown in Figure 4. The total spin moment in **1-Cr** (0.04 μ_B_ per magnetic atom, with a canting angle of about 0.63°) is significantly smaller than the previous estimate (1 μ_B_, 14.5°).^12^ The present calculations emphasizing a very small canting angle are more consistent with the experiment in this work that could not see the WFM (Figure 3(c)).

Here, we adopt the same classical spin model introduced in ref (12). The model comprises the competition between a standard Heisenberg term , with an intralayer ferromagnetic exchange *J*_*ab*_ < 0, an interlayer antiferromagnetic exchange *J*_*c*_ > 0, and a site-dependent MSIA term. The latter can be written in a local reference frame defined by M–X bonds as *H*_sia_ = *E* ∑_*i*_ [(***S***_*i*_·***e***_*i*_^s^)^2^ – (***S***_*i*_·***e***_*i*_^l^)^2^] + *D*∑_*i*_(***S***_*i*_·***e***_*i*_^m^)^2^, with *E* and *D* denoting the principal values of the MSIA tensor and ***e***_*i*_^l^, ***e***_*i*_^m^, and ***e***_*i*_^s^ denoting the long, medium, and short M–X bonds of the JT-distorted MX_6_ octahedra. We estimated the model parameters from the total-energy mapping of DFT computations with different collinear and canted magnetic configurations (see Section S4 for details). When comparing with previous estimates^12^ obtained within a bare GGA approach, we observe a substantial reduction of both *J*_*ab*_ and *J*_*c*_ exchanges (as expected by the inclusion of the *U* correction) and, most prominently, of the MSIA parameter *E* (see Table 1).

**Table 1:** Parameters of the Spin Model^a^

	(*U*, *J*)	*J*_*c*_	*J*_*ab*_	*D*	*E*	θ_t_	
no *U* (ref (12))	(0, 0)	0.824	–0.453	0.113	0.745	30.8	
no *U* (this work)	(0, 0)	0.837	–0.459	0.092	0.035 (0.036)	33.6 (31.9)	
set 1	(2.5, 0.5)	0.475	–0.111	0.115	0.038 (0.041)	33.8 (31.9)	
set 2	(3.0, 1.0)	0.453	–0.042	0.149	0.059 (0.062)	33.8 (31.9)	

a Model parameters (all couplings in meV, tilting angle θ_t_ in degrees) used in the Monte Carlo simulations for **1-Cr**, with and without the +*U* + *J* corrections. The parameters in the previous work^12^ are also shown for comparison. Notice that the estimate for the angle θ_t_ has been obtained from magnetic anisotropy energies.^12^ The fact that it is the same in the two sets is a nontrivial result. Such an estimate is in good agreement also with the structural tilting angle (given in brackets). The values in parentheses for the parameter *E* are the estimates obtained by using the structural tilting angle θ_t_, showing small deviations that are found not to affect the results significantly.

**Figure 5:**
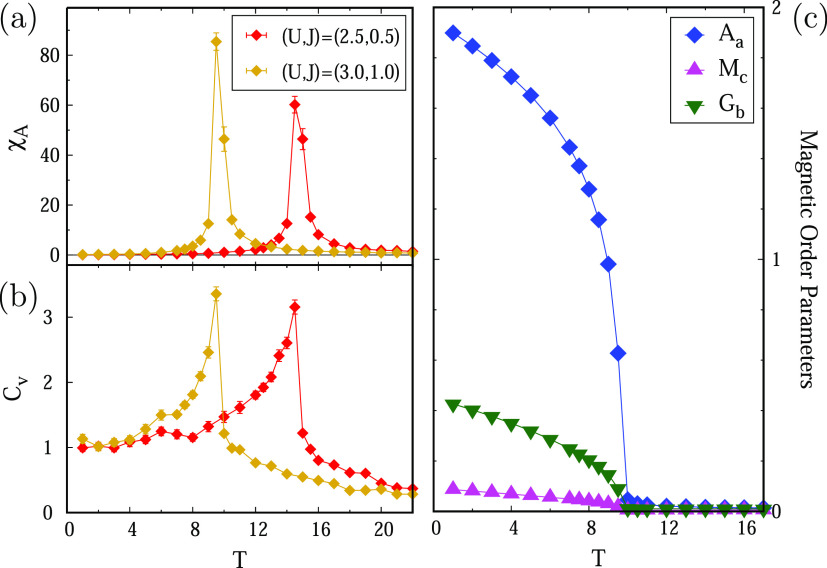
Monte Carlo calculations. (a) Susceptibility associated with the primary order parameter A-AFM and (b) specific heat as a function of temperature and calculated from two sets of model parameters extracted from DFT + *U* + *J* computations. Both quantities display sharp peaks, signaling transition temperatures in a range between 9.5 and 14.5 K. Panel (c) displays the evolution of order parameters *A*_*a*_, *G*_*b*_, and *M*_*c*_ below the transition temperature for the set of parameters corresponding to (*U*, *J*) = (3, 1) eV.

In addition, we performed Monte Carlo simulations using the new parameters (see Section S5 for further details). Results are summarized in Figure 5, where the evolution with the temperature of specific heat, order parameters, and susceptibility of the primary A-AFM order parameter are shown. The magnetic transition is estimated between 9.5 and 14.5 K, in fairly good agreement with the experimental data (∼8 K). The primary order parameter is A-AFM, as shown by the sharp peak in the susceptibility, aligned along the *a*-axis. A WFM aligned along the *c*-axis also arises under the *T*_c_ as a secondary order parameter, consistently with the prediction by DFT,^12^ even though it is strongly renormalized, while the other competing secondary parameter, corresponding to a G-AFM canting parallel to the *b*-axis, is found to be substantial (see Section S4). The dominating antiferromagnetic interactions can also be deduced by the powder-average magnetic susceptibility, as shown in the inset of Figure 3(a) (see Figure S9 for the full data), and they are again in good agreement with the experimental data.

In this study, we calculate and model the orbital magnetic moment in **1-Cr/Cu**, which was neglected in previous studies^9,12^ in more detail and is not generally accounted for in the studies of metal–organic formates. Here, we show that it may have a non-negligible contribution to the total magnetic moment. Generally, the magnetic moment has spin and orbital contributions. For a transition metal ion in an octahedral environment, the orbital magnetic moment is quenched when the *t*_2*g*_ d-orbitals are fully or half-filled. However, the presence of SOC can still induce a small orbital magnetic moment.^65^ In the case where the spin contribution to the ferromagnetic component is much smaller than 1 μ_B_ per magnetic ion, such as the case of WFM with a tiny canting angle, the orbital contribution to the ferromagnetic component may become significant to it. Figure 4(a,b) shows the calculated spin and orbital magnetic moment of **1-Cu** along the *a* direction and **1-Cr** along the *c* direction, respectively. Indeed, we found that in **1-Cu**, the orbital ferromagnetic component is collinear and comparable in magnitude to the spin magnetic moment, the latter of which is not commonly expected for the orbital-quenched *d*^9^ case. In the range of large |λ|, the orbital contribution (0.070 μ_B_ per unit cell at λ = 1) to the ferromagnetic moment is larger than the spin contribution (0.043 μ_B_). On the other hand, for **1-Cr**, the contribution of the orbital moment to the ferromagnetic net moment (−0.024 μ_B_) is much smaller in comparison with the contribution from the spin moment (0.172 μ_B_) and they are anticollinear. Notably, the experimental effective magnetic moment for **1-Cr** is lower than the spin-only magnetic moment, while that of **1-Cu** is notably higher than its spin-only moment.^25^ These are reminiscent of Hund’s third rule of the atomic limit: the spin and orbital moments are anticollinear for the less-than-half-filled case (**1-Cr**), but they are aligned for the more-than-half-filled case (**1-Cu**). In the next section, we define a model of the orbital angular momentum in **1-Cr/Cu** and its interplay with JT distortion/orbital ordering, which perfectly accounts for the observed trends.

### Model for Orbital Magnetic Moment in **1-Cr**/Cu

In order to explain the orbital magnetic moment in **1-Cr/Cu**, we introduce a model based on perturbation theory and JT effective Hamiltonian within a single-ion description, which is an improvement of the previous work.^12^ The perturbation approach for the orbital angular momentum and MSIA corresponds to the Bruno theory,^53,54^ but we ignore the *k*-space dispersion for simplicity. The SOC Hamiltonian is written as *H*_SOC_ = ζ**S**·**L**, where **S** and **L** are spin and orbital angular momentum operators, respectively. We will consider only d-orbitals here.^55^

In the O_6_ octahedron cage, the crystal field splits the d-orbitals into lower energy *t*_2*g*_ orbitals (d_*yz*_, d_*zx*_, and d_*xy*_) and higher energy *e*_*g*_ orbitals (d_*x*^2^–*y*^2^_, d_*z*^2^_). In addition, the JT effect splits the degeneracy of *e*_*g*_ orbitals by deforming the O_6_ cage.^50,56^ This deformation is represented by two distortion modes, *Q*_2_ = (1/√2)(*l*_*x*_ – *l*_*y*_) and *Q*_3_ = (1/√6)(2*l*_*z*_ – *l*_*x*_ – *l*_*y*_), where the *l*_*i*_ means the distance from the center to the oxygen on the *i*-axis. The JT-distorted structure is expressed with the JT phase θ_JT_ as |θ_JT_⟩ = cos θ_JT_|*Q*_3_⟩ + sin θ_JT_|*Q*_2_⟩ and tan θ_JT_ = *Q*_2_/*Q*_3_. The JT effective Hamiltonian taking the *e*_*g*_ orbitals as a basis is given by
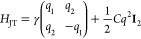
1where *q*_1_ = *q* cos θ_JT_, *q*_2_ = *q* sin θ_JT_, and **I**_2_ is the 2 × 2 identity matrix.^50,57−59^ The energy eigenvalues are  and eigenstates are
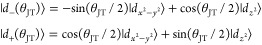
2These unitary rotated *e*_*g*_ orbitals rewrite the orbital angular momentum operator and, consequently, the SOC Hamiltonian *H*_SOC_ (see Section S7 for details).

The perturbation theory is applied to obtain the orbital angular momentum induced by SOC. The d-orbitals with the “JT-rotated” *e*_*g*_ orbitals (eq 2) are taken as the unperturbed basis. Then, the orbital angular momenta are obtained as expectation values of the orbital angular momentum operators defined with the JT-rotated *e*_*g*_ orbitals up to the first order in ζ (see Section S8 for the details). As a result, the orbital angular moments of the *d*^4^ (**1-Cr**) configuration are
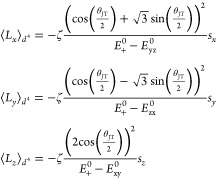
3for each *x*, *y*, and *z* component, where **ŝ** = (*s*_*x*_, *s*_*y*_, *s*_*z*_) is the local spin direction with respect to the local orbital coordinates aligned to the octahedron. We adopt the atomic units in which ℏ = 1. For the *d*^9^ configuration (**1-Cu**), ⟨*L*_*i*_⟩_*d*^9^_ = −⟨*L*_*i*_⟩_*d*^4^_. It is noteworthy that the resultant local orbital moment is not always parallel to the spin. The deviation is explicitly determined by θ_JT_.

The orbital angular momentum formula, eq 3, can be used for the orbital magnetic moment by simply replacing the angular momentum with the magnetic moment in the Bohr magneton μ_B_ unit for both the spin and orbital as we use the atomic units. As stated in the previous section, two magnetic groups, *Pn*′*a*′2_1_ and *Pna*′2_1_′, are considered for both **1-Cr** (*d*^4^) and **1-Cu** (*d*^9^). For simplicity, we will ignore spin canting in the following analysis. Once the local magnetic moment at a reference Cr/Cu site is obtained by eq 3, the magnetic space group symmetry determines the magnetic moments at other sites according to the rules listed in Table 2. For the details, see Section S9 of the SI.

**Table 2:** Symmetry Operations in *Pna*′2_1_′ and *Pn*′*a*′2_1_^a^

*Pna*′2_1_′		*Pn*′*a*′2_1_	
op.	*L⃗*	op.	*L⃗*
1	(*L*_*a*_, *L*_*b*_, *L*_*c*_)	1	(*L*_*a*_, *L*_*b*_, *L*_*c*_)
*n*	(*L*_*a*_, −*L*_*b*_, −*L*_*c*_)	*n*′	(−*L*_*a*_, *L*_*b*_, *L*_*c*_)
*a*′	(*L*_*a*_, −*L*_*b*_, *L*_*c*_)	*a*′	(*L*_*a*_, −*L*_*b*_, *L*_*c*_)
2_1_′	(*L*_*a*_, *L*_*b*_, −*L*_*c*_)	2_1_	(−*L*_*a*_, −*L*_*b*_, *L*_*c*_)

a Symmetry operations and the transformation rules of the magnetic moment (*L⃗*) in the magnetic space group *Pna*′2_1_′ and *Pn*′*a*′2_1_. 1 represents the identity operation. Subscripts *a*, *b*, and *c* are the crystallographic axes.

For the *d*^4^ configuration (Cr^2+^) with *Pn*′*a*′2_1_ symmetry, the total orbital moment is
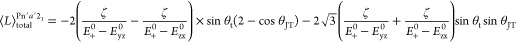
4along the *c*-axis, where θ_t_ is the tilting angle of the O_6_ octahedron from the *c*-axis. Since the difference between the *t*_2*g*_ orbitals *E*_*yz*_^0^ and *E*_*zx*_^0^ is smaller than the crystal field splitting, the first term is small compared to the second term. Moreover, ⟨*L*⟩_total_ vanishes when θ_JT_ = π or λ = 0, since *l*_*x*_ ≈ *l*_*y*_ leads to *E*_*yz*_^0^ ≈ *E*_*zx*_^0^. It is consistent with the first-principles results that the total ferromagnetic moment vanishes at λ = 0. By introducing the approximation *E*_*yz*_^0^ = *E*_*zx*_^0^ = *E*_*xy*_^0^ ≡ *E*_*t*_2*g*__^0^, the orbital magnetic moment can be simplified as
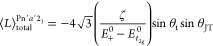
5Interestingly, for the *d*^4^(Cr^2+^) with *Pna*′2_1_′ symmetry, the same total orbital magnetic moment formula is obtained, ⟨*L*⟩_total_^*Pna*^′^2_1_^′^^ = ⟨*L*⟩_total_^*Pn*^′^*a*^′^2_1_^, except that the direction is along the *a*-axis. Therefore, the same arguments are also valid, leading to the same simplified form of eq 5. For the *d*^9^ configuration (Cu^2+^), the sign of the orbital magnetic moment is inverted in both magnetic groups.

**Figure 6:**
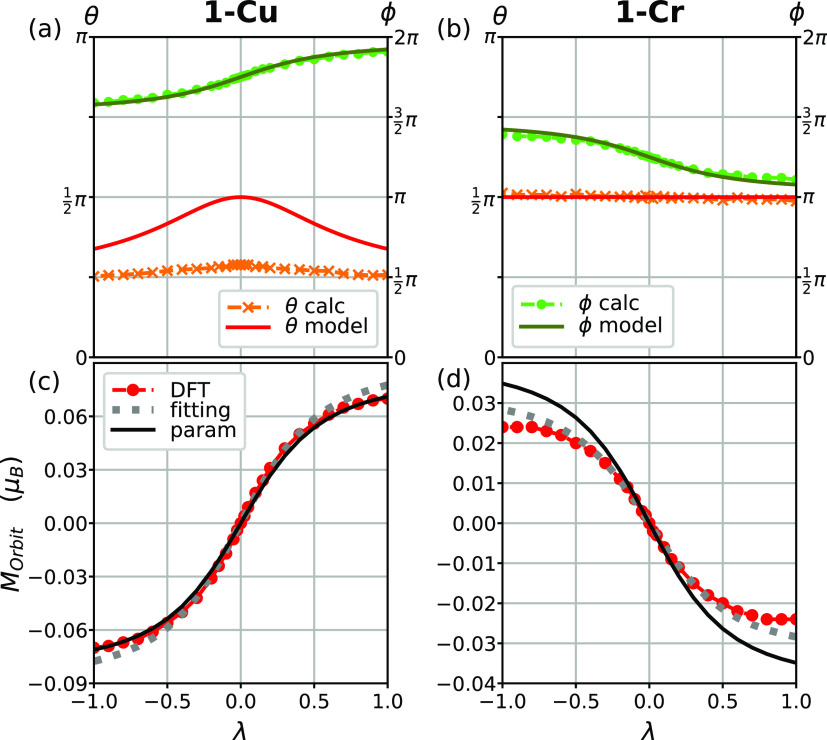
Direction of the local orbital magnetic moment of reference ions of (a) **1-Cu** and (b) **1-Cr** in their local spherical coordinates (θ: polar/ϕ: azimuthal) obtained from DFT and the model. The total orbital magnetic moments of (c) **1-Cu** and (d) **1-Cr** obtained from DFT and the model. Model values in panels (c, d) are either fitted to the DFT results (gray dotted line) or evaluated from reasonable physical parameters (black solid line).

As a preliminary step for the comparison between the DFT results and the predictions from the model, we parametrize the JT phase of a reference Cr/Cu ion as a function of λ, tan(π–θ_JT_)/tan(π–θ_JT,λ=1_) = λ, where θ_JT,λ=1_ is the JT phase at λ = 1 (see Section S9 for the details). For simplicity, let us consider θ_JT,λ=1_ = 2π/3. The JT phase becomes θ_JT_ = π – tan^–1^(√3λ). As a result, the simplified orbital magnetic moment, eq 5, can be written in terms of λ.
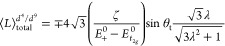
6Ignoring the JT phase dependency of *E*_*i*_^0^’s, we define the λ independent factor of this expression as *A* = ∓4√3(ζ/(*E*_+_^0^ – *E*_*t*_2*g*__^0^))sin θ_t_. To check the validity of our approach, we compared the model with the DFT values by fitting the single parameter *A*. The fitted *A* values are 0.090 for **1-Cu** and −0.033 for **1-Cr**. In addition, the *A* values are estimated from physical parameters as 0.082 for **1-Cu** and −0.040 for **1-Cr**, which are in good agreement with the fitted values (see Section S9). As shown in Figure 6(c,d), the model with these *A* values well explains the DFT results.

As a further step, let us consider the direction of the local orbital magnetic moment of a reference Cu and Cr ion expected from eq 3, as shown in Figure 6(a,b) in the spherical coordinates with respect to their local orbital-axes, respectively. The directions from DFT are shown together for comparison. Except for the deviation in the polar angle θ of **1-Cu**, the model well predicts the orbital magnetic moment direction. The deviation may originate from oversimplification of the model, such as ignoring the θ_JT_-dependence of orbital energies, degeneracy, and higher orders in ζ.

Interestingly, the model accounts well for the finite orbital magnetic moment, although we assumed the uncanted spin configuration in which the total spin moment vanishes. This is possible because the local spin and orbital moments are not parallel in **1-Cr/Cu**. The orbital moment rotates depending on the JT phase in spite of the fixed spin direction. The inclusion of the small spin canting would not significantly alter the predicted orbital moment (∼1%). In other words, the orbital moment is insensitive to a moderate spin fluctuation, i.e., the orbital moment is robust. Moreover, the orbital magnetic moment is linear to the SOC strength ζ, whereas the MSIA is second-order (Section S10). These properties provide a robust justification of WFM in terms of the total magnetic moment (spin + orbital) for **1-Cu** and also emphasize the role of the JT effect on it. Our prediction might be confirmed by an X-ray magnetic circular dichroism experiment,^60^ which can measure the orbital magnetic moment.

**Figure 7:**
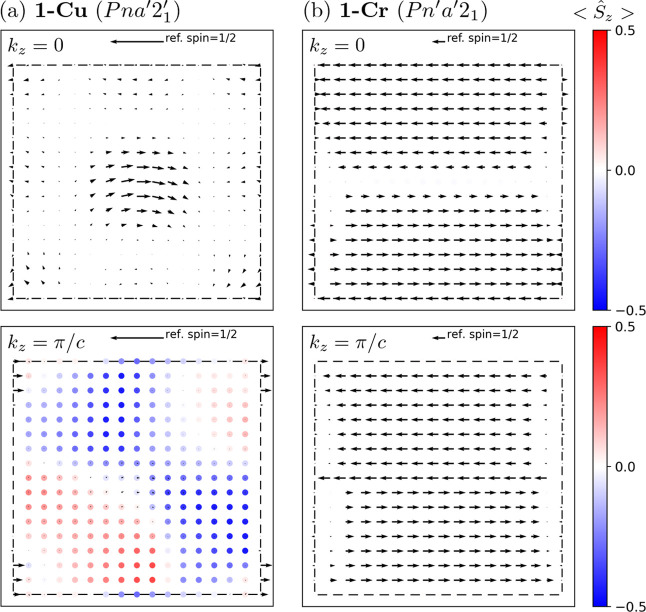
Spin textures of (a) **1-Cu** and (b) **1-Cr** at the *k*_*z*_ = 0 (upper panels) and *k*_*z*_ = π/*c* (lower panels) planes. The in-plane *x* and *y* components are represented by arrows of which the length is the in-plane magnitude with respect to the reference spin 1/2 above the figure. The *z* components are represented by a colormap of dots. The inner boundary of each figure coincides with the Brillouin zone boundary.

Finally, we note that the complex interplay between the SOC and JT distortion may induce unusual spin and orbital textures in the *k*-space. The spin texture (**s**_**k***n*_ = ⟨ψ_k*n*_|**S**|ψ_**k***n*_⟩, where *n* is a band index) of the highest occupied band of each compound at *k*_*z*_ = 0 and *k*_*z*_ = π/*c* planes are shown in Figure 7. In **1-Cu**, a “curly” in-plane texture appears around the center at the *k*_*z*_ = 0 plane. On the other hand, an irregular texture mostly aligned to the *z*-direction can be seen at the *k*_*z*_ = π/*c* plane. In **1-Cr**, persistent-type spin textures^61^ appear for both the *k*_*z*_ = 0 and *k*_*z*_ = π/*c* planes. Since the orbital magnetic moment is non-negligible in **1-Cu**, it is worth investigating the orbital texture in **1-Cr/Cu**. For example, we found a Dresselhaus-type^62^ orbital texture in **1-Cr** (see Section S11 in the SI).

## Conclusions

In this work, we reported for the first time the synthesis of **1-Cr**, a missing member of the [C(NH_2_)_3_]M(HCOO)_3_ family. We identified that **1-Cr** has a perovskite-type JT-distorted polar *Pna*2_1_ structure as initially predicted, as well as an AFM ordering below 8 K. We provided an improved understanding of the magnetic properties of **1-Cr/Cu** by the first-principles calculation and model studies, which provide a better estimate of the *T*_c_ by including the on-site Coulomb energy corrections in DFT. The revised magnetic moment estimation is also more consistent with the experiment. In addition, new calculations suggested the potential importance of the orbital magnetic moment in **1-Cr/Cu**, i.e., the contribution of the orbital magnetic moment to the net ferromagnetic moment is even larger than the spin contribution in **1-Cu**. We revealed that the orbital magnetic moment is robust and explicitly coupled with the JT distortion by the model study. This approach can generally be considered in the *d*^4^/*d*^9^ JT systems.

## Supporting Information Available

Additional sample preparation details; single crystal diffraction measurements and analysis; symmetry analysis of the magnetic ground state; spin model; Monte Carlo results; DFT parameter dependence of magnetic moments; matrix representations of the spin–orbit coupling; calculation of the perturbed orbital angular momentum; details in the model analysis for **1-Cr**/**Cu**; the second-order energy correction term in the perturbation theory; spin and orbital textures in *k*-space (PDF)

## References

[ref1] Furukawa H.; Cordova K. E.; O’Keeffe M.; Yaghi O. M. The Chemistry and Applications of Metal–Organic Frameworks. Science 2013, 341, 1230444 10.1126/science.1230444. 23990564

[ref2] Suh M. P.; Park H. J.; Prasad T. K.; Lim D.-W. Hydrogen Storage in Metal–Organic Frameworks. Chem. Rev. 2012, 112, 782–835. 10.1021/cr200274s. 22191516

[ref3] Cheetham A. K.; Rao C. N. R. There’s Room in the Middle. Science 2007, 318, 58–59. 10.1126/science.1147231. 17916720

[ref4] Wang X.-Y.; Gan L.; Zhang S.-W.; Gao S. Perovskite-like Metal Formates with Weak Ferromagnetism and as Precursors to Amorphous Materials. Inorg. Chem. 2004, 43, 4615–4625. 10.1021/ic0498081. 15257590

[ref5] Ye Q.; Song Y.-M.; Wang G.-X.; Chen K.; Fu D.-W.; Chan P. W. H.; Zhu J.-S.; Huang S. D.; Xiong R.-G. Ferroelectric Metal–Organic Framework with a High Dielectric Constant. J. Am. Chem. Soc. 2006, 128, 6554–6555. 10.1021/ja060856p. 16704244

[ref6] Van Aken B. B.; Palstra T. T.; Filippetti A.; Spaldin N. A. The origin of ferroelectricity in magnetoelectric YMnO_3_. Nat. Mater. 2004, 3, 164–170. 10.1038/nmat1080. 14991018

[ref7] Cheong S.-W.; Mostovoy M. Multiferroics: a magnetic twist for ferroelectricity. Nat. Mater. 2007, 6, 13–20. 10.1038/nmat1804. 17199121

[ref8] Malashevich A.; Vanderbilt D. First Principles Study of Improper Ferroelectricity in TbMnO_3_. Phys. Rev. Lett. 2008, 101, 037210 10.1103/PhysRevLett.101.037210. 18764292

[ref9] Stroppa A.; Jain P.; Barone P.; Marsman M.; Perez-Mato J. M.; Cheetham A. K.; Kroto H. W.; Picozzi S. Electric Control of Magnetization and Interplay between Orbital Ordering and Ferroelectricity in a Multiferroic Metal–Organic Framework. Angew. Chem., Int. Ed. 2011, 50, 5847–5850. 10.1002/anie.201101405. 21618371

[ref10] Picozzi S.; Stroppa A. Advances in ab-initio theory of multiferroics: Materials and mechanisms: modelling and understanding. Eur. Phys. J. B 2012, 85, 240 10.1140/epjb/e2012-30124-1.

[ref11] Zhang W.; Xiong R.-G. Ferroelectric Metal–Organic Frameworks. Chem. Rev. 2012, 112, 1163–1195. 10.1021/cr200174w. 21939288

[ref12] Stroppa A.; Barone P.; Jain P.; Perez-Mato J. M.; Picozzi S. Hybrid Improper Ferroelectricity in a Multiferroic and Magnetoelectric Metal–Organic Framework. Adv. Mater. 2013, 25, 2284–2290. 10.1002/adma.201204738. 23386395

[ref13] Di Sante D.; Stroppa A.; Jain P.; Picozzi S. Tuning the Ferroelectric Polarization in a Multiferroic Metal–Organic Framework. J. Am. Chem. Soc. 2013, 135, 18126–18130. 10.1021/ja408283a. 24191632

[ref14] Tian Y.; Stroppa A.; Chai Y.-S.; Barone P.; Perez-Mato M.; Picozzi S.; Sun Y. High-Temperature Ferroelectricity and Strong Magnetoelectric Effects in a Hybrid Organic-Inorganic Perovskite Framework. Phys. Status Solidi RRL 2015, 9, 62–67. 10.1002/pssr.201409470.

[ref15] Gómez-Aguirre L. C.; Pato-Doldán B.; Stroppa A.; Yang L.-M.; Frauenheim T.; Mira J.; Vilar S. Y.; Artiaga R.; Castro-García S.; Sánchez-Andújar M.; Señarís-Rodríguez M. A. Coexistence of Three Ferroic Orders in the Multiferroic Compound [(CH_3_)_4_N][Mn(N_3_)_3_] with Perovskite-Like Structure. Chem. - Eur. J. 2016, 22, 7863–7870. 10.1002/chem.201503445. 27072487

[ref16] Evans N. L.; Thygesen P. M. M.; Boström H. L. B.; Reynolds E. M.; Collings I. E.; Phillips A. E.; Goodwin A. L. Control of Multipolar and Orbital Order in Perovskite-like [C(NH_2_)_3_]Cu_x_Cd_1–x_(HCOO)_3_ Metal–Organic Frameworks. J. Am. Chem. Soc. 138, 9393–9396 10.1021/jacs.6b05208. 27414161

[ref17] Fan F.-R.; Wu H.; Nabok D.; Hu S.; Ren W.; Draxl C.; Stroppa A. Electric-Magneto-Optical Kerr Effect in a Hybrid Organic–Inorganic Perovskite. J. Am. Chem. Soc. 2017, 139, 12883–12886. 10.1021/jacs.7b04911. 28853870

[ref18] Saines P. J.; Bristowe N. C. Probing magnetic interactions in metal–organic frameworks and coordination polymers microscopically. Dalton Trans. 2018, 47, 13257–13280. 10.1039/C8DT02411A. 30112541

[ref19] Ma Y.; Sun Y. Multiferroic and thermal expansion properties of metal-organic frameworks. J. Appl. Phys. 2020, 127, 080901 10.1063/1.5137819.

[ref20] Kanižaj L.; Barišić D.; Torić F.; Pajić D.; Molčanov K.; Šantić A.; Lončarić I.; Jurić M. Structural, Electrical, and Magnetic Versatility of the Oxalate-Based [CuFe] Compounds Containing 2,2’:6’,2”-Terpyridine: Anion-Directed Synthesis. Inorg. Chem. 2020, 59, 18078–18089. 10.1021/acs.inorgchem.0c02548. 33289548

[ref21] Šenjug P.; Dragović J.; Kalanj M.; Torić F.; Rubčić M.; Pajić D. Magnetic behaviour of (C_2_H_5_NH_3_)_2_CuCl_4_ type multiferroic. J. Magn. Magn. Mater. 2019, 479, 144–148. 10.1016/j.jmmm.2019.02.020.

[ref22] Yang Z.; Cai G.; Bull C. L.; Tucker M. G.; Dove M. T.; Friedrich A.; Phillips A. E. Hydrogen-bond-mediated structural variation of metal guanidinium formate hybrid perovskites under pressure. Philos. Trans. R. Soc., A 2019, 377, 20180227 10.1098/rsta.2018.0227. PMC6562345 31130096

[ref23] Gonçalves J. N.; Phillips A. E.; Li W.; Stroppa A. First-Principles Study of Structure and Magnetism in Copper(II)-Containing Hybrid Perovskites. Crystals 2020, 10, 1129 10.3390/cryst10121129.

[ref24] Kang S.; Yu J. Electronic structure and magnetic properties of transition metal kagome metal–organic frameworks. Phys. Chem. Chem. Phys. 2022, 24, 22168–22180. 10.1039/D2CP02612K. 36093573

[ref25] Hu K.-L.; Kurmoo M.; Wang Z.; Gao S. Metal–Organic Perovskites: Synthesis, Structures, and Magnetic Properties of [C(NH _2_)_3_][M^II^(HCOO)_3_] (M = Mn, Fe, Co, Ni, Cu, and Zn; C(NH_2_)_3_ = Guanidinium). Chem. Eur. J. 2009, 15, 12050–12064. 10.1002/chem.200901605. 19774570

[ref26] Benedek N. A.; Fennie C. J. Hybrid Improper Ferroelectricity: A Mechanism for Controllable Polarization-Magnetization Coupling. Phys. Rev. Lett. 2011, 106, 107204 10.1103/PhysRevLett.106.107204. 21469829

[ref27] Rondinelli J. M.; Fennie C. J. Octahedral Rotation-Induced Ferroelectricity in Cation Ordered Perovskites. Adv. Mater. 2012, 24, 1961–1968. 10.1002/adma.201104674. 22488734

[ref28] Benedek N. A.; Rondinelli J. M.; Djani H.; Ghosez P.; Lightfoot P. Understanding ferroelectricity in layered perovskites: new ideas and insights from theory and experiments. Dalton Trans. 2015, 44, 10543–10558. 10.1039/C5DT00010F. 25687622

[ref29] Boström H. L. B.; Senn M. S.; Goodwin A. L. Recipes for improper ferroelectricity in molecular perovskites. Nat. Commun. 2018, 9, 2380 10.1038/s41467-018-04764-x. 29915202 PMC6006342

[ref30] Ai Y.; Sun R.; Liao W.-Q.; Song X.-J.; Tang Y.-Y.; Wang B.-W.; Wang Z.-M.; Gao S.; Xiong R.-G. Unprecedented Ferroelectricity and Ferromagnetism in a Cr^2+^ Based Two-Dimensional Hybrid Perovskite. Angew. Chem., Int. Ed. 2022, 61, e202206034 10.1002/anie.202206034. 35604204

[ref31] Ai Y.; Sun R.; Zeng Y.-L.; Liu J.-C.; Tang Y.-Y.; Wang B.-W.; Wang Z.-M.; Gao S.; Xiong R.-G. Coexistence of magnetic and electric orderings in a divalent Cr^2+^-based multiaxial molecular ferroelectric. Chem. Sci. 2021, 12, 9742–9747. 10.1039/D1SC01871J. 34349946 PMC8293986

[ref32] Brauer G. Handbook of Preparative Inorganic Chemistry, 2nd ed.; Academic Press, 1963.

[ref33] Howard C. J.; Hunter B. A. A Computer Program for Rietveld Analysis of X-ray and Neutron Powder Diffraction Patterns 1998.

[ref34] Kresse G.; Hafner J. *Ab initio* molecular dynamics for liquid metals. Phys. Rev. B 1993, 47, 558–561. 10.1103/PhysRevB.47.558. 10004490

[ref35] Kresse G.; Hafner J. *Ab initio* molecular-dynamics simulation of the liquid-metal–Amorphous-semiconductor transition in germanium. Phys. Rev. B 1994, 49, 14251–14269. 10.1103/PhysRevB.49.14251. 10010505

[ref36] Kresse G.; Furthmüller J. Efficiency of ab-initio total energy calculations for metals and semiconductors using a plane-wave basis set. Comput. Mater. Sci. 1996, 6, 15–50. 10.1016/0927-0256(96)00008-0. 9984901

[ref37] Kresse G.; Furthmüller J. Efficient iterative schemes for *ab initio* total-energy calculations using a plane-wave basis set. Phys. Rev. B 1996, 54, 11169–11186. 10.1103/PhysRevB.54.11169. 9984901

[ref38] Perdew J. P.; Burke K.; Ernzerhof M. Generalized Gradient Approximation Made Simple. Phys. Rev. Lett. 1996, 77, 3865–3868. 10.1103/PhysRevLett.77.3865. 10062328

[ref39] Kresse G.; Joubert D. From ultrasoft pseudopotentials to the projector augmented-wave method. Phys. Rev. B 1999, 59, 1758–1775. 10.1103/PhysRevB.59.1758.

[ref40] Capillas C.; Tasci E. S.; Flor G. d. l.; Orobengoa D.; Perez-Mato J. M.; Aroyo M. I. A new computer tool at the Bilbao Crystallographic Server to detect and characterize pseudosymmetry. Z. Kristallogr. 2011, 226, 186–196. 10.1524/zkri.2011.1321.

[ref41] Liechtenstein A. I.; Anisimov V. I.; Zaanen J. Density-functional theory and strong interactions: Orbital ordering in Mott-Hubbard insulators. Phys. Rev. B 1995, 52, R5467 10.1103/PhysRevB.52.R5467. 9981806

[ref42] Brese N. E.; O’Keeffe M. Bond-valence parameters for solids. Acta Cryst. B 1991, 47, 192–197. 10.1107/S0108768190011041.

[ref43] Fackler J. P. J.; Holah D. G. Properties of Chromium(II) Complexes. I. Electronic Spectra of the Simple Salt Hydrates. Inorg. Chem. 1965, 4, 954–958. 10.1021/ic50029a006.

[ref44] Aakesson R.; Pettersson L. G. M.; Sandstroem M.; Wahlgren U. Theoretical calculations of the Jahn-Teller effect in the hexahydrated Copper(II), chromium(II), and manganese(III) ions, hexaaquacopper(2+), hexaaquachromium(2+) and hexaaquamanganese(3+), and comparisons with the hexahydrated copper(I), chromium(III), and manganese(II) clusters. J. Phys. Chem. A 1992, 96, 150–156. 10.1021/j100180a031.

[ref45] Collings I. E.; Hill J. A.; Cairns A. B.; Cooper R. I.; Thompson A. L.; Parker J. E.; Tang C. C.; Goodwin A. L. Compositional dependence of anomalous thermal expansion in perovskite-like ABX_3_ formates. Dalton Trans. 2016, 45, 4169–4178. 10.1039/C5DT03263F. 26477747

[ref46] Burley L. G.; Beecham-Lonsdale J. H.; Srivastava A. K.; Collings I. E.; Saines P. J. Enhancing the chemical flexibility of hybrid perovskites by introducing divalent ligands. Dalton Trans. 2021, 50, 5437–5441. 10.1039/D1DT00878A. 33908998

[ref47] Vasiliev A. N.; Volkova O. S.; Hammer E.; Glaum R.; Broto J.-M.; Millot M.; Nénert G.; Liu Y. T.; Lin J.-Y.; Klingeler R.; Abdel-Hafiez M.; Krupskaya Y.; Wolter A. U. B.; Büchner B. Weak ferrimagnetism and multiple magnetization reversal in α-Cr_3_(PO_4_)_2_. Phys. Rev. B 2012, 85, 014415 10.1103/PhysRevB.85.014415.

[ref48] Tezuka K.; Shan Y. J.; Imoto H.; Ohoyama K. Crystal and magnetic structures of Y_2_CrS_4_. J. Phys. Chem. Solids 2007, 68, 2133–2137. 10.1016/j.jpcs.2007.08.016.

[ref49] Goodenough J. B. Magnetism and the Chemical Bond; R. E. Krieger Pub. Co, 1976.

[ref50] Khomskii D. I. Transition Metal Compounds; Cambridge University Press, 2014.

[ref51] Šenjug P.; Dragović J.; Torić F.; Lončarićć I.; Despoja V.; Smokrović K.; Topić E.; Dilović I.; Rubčić M.; Pajić D. Magnetoelectric Multiferroicity and Magnetic Anisotropy in Guanidinium Copper(II) Formate Crystal. Materials 2021, 14, 1730 10.3390/ma14071730. 33916071 PMC8036506

[ref52] Moriya T. Theory of Magnetism of NiF_2_. Phys. Rev. 1960, 117, 635–647. 10.1103/PhysRev.117.635.

[ref53] Bruno P. Tight-binding approach to the orbital magnetic moment and magnetocrystalline anisotropy of transition-metal monolayers. Phys. Rev. B 1989, 39, 865–868. 10.1103/PhysRevB.39.865. 9947253

[ref54] Blanco-Rey M.; Cerdá J. I.; Arnau A. Validity of perturbative methods to treat the spin–orbit interaction: application to magnetocrystalline anisotropy. New J. Phys. 2019, 21, 073054 10.1088/1367-2630/ab3060.

[ref55] Takayama H.; Bohnen K.-P.; Fulde P. Magnetic surface anisotropy of transition metals. Phys. Rev. B 1976, 14, 2287–2295. 10.1103/PhysRevB.14.2287.

[ref56] Jahn H.; Teller E. Stability of polyatomic molecules in degenerate electronic states-I Orbital degeneracy. Proc. R. Soc. London, Ser. A 1937, 161, 220–235. 10.1098/rspa.1937.0142.

[ref57] Bersuker I. B. Modern Aspects of the Jahn-Teller Effect Theory and Applications To Molecular Problems. Chem. Rev. 2001, 101, 1067–1114. 10.1021/cr0004411. 11709858

[ref58] Pavarini E.; Koch E.; Anders F.; Jarrell M. Correlated Electrons: from Models to Materials. In Reihe Modeling and Simulation; Forschungszentrum Jülich: Zentralbibliothek, Verlag, 2012.

[ref59] Stroppa A.; Barone P.; Di Sante D.; Cuoco M.; Picozzi S.; Whangbo M.-H. Analogies between Jahn-Teller and Rashba spin physics. Int. J. Quantum Chem. 2016, 116, 1442–1450. 10.1002/qua.25209.

[ref60] Thole B. T.; Carra P.; Sette F.; van der Laan G. X-ray circular dichroism as a probe of orbital magnetization. Phys. Rev. Lett. 1992, 68, 1943–1946. 10.1103/PhysRevLett.68.1943. 10045260

[ref61] Tao L. L.; Tsymbal E. Y. Persistent spin texture enforced by symmetry. Nat. Commun. 2018, 9, 2763 10.1038/s41467-018-05137-0. 30018283 PMC6050308

[ref62] Winkler R. Spin-Orbit Coupling Effects in Two-Dimensional Electron and Hole Systems. In Springer Tracts in Modern Physics; Springer, 2003; pp 69–129.

[ref63] Dzyaloshinsky I. A thermodynamic theory of “weak” ferromagnetism of antiferromagnetics. J. Phys. Chem. Solids 1958, 4, 241–255. 10.1016/0022-3697(58)90076-3.

[ref64] Moriya T. Anisotropic Superexchange Interaction and Weak Ferromagnetism. Phys. Rev. 1960, 120, 91–98. 10.1103/PhysRev.120.91.

[ref65] Dai D.; Xiang H.; Whangbo M.-H. Effects of Spin-Orbit Coupling on Magnetic Properties of Discrete and Extended Magnetic Systems. J. Comput. Chem. 2008, 29, 2187–2209. 10.1002/jcc.21011. 18484639

