# Supplementary material for: Synthesis and Magnetic Properties of the Multiferroic [C(NH2)3]Cr(HCOO)3 Metal–Organic Framework: The Role of Spin–Orbit Coupling and Jahn–Teller Distortions
Source: Inorg Chem. 2023 Oct 11;62(42):17299–309. doi: 10.1021/acs.inorgchem.3c02557 (PMC10598855; doi:10.1021/acs.inorgchem.3c02557)
Supplement: Supplementary file 1 — ic3c02557_si_001.pdf [file ic3c02557_si_001.pdf]

Supporting Information for "Synthesis and Magnetic Properties of the Multiferroic $[C(NH_2)_3]Cr(HCOO)_3$ Metal-Organic Framework: The Role of Spin-Orbit Coupling and Jahn-Teller Distortions"

Kunihiro Yananose,[†,‡,⊥] Ewan R. Clark,[¶,⊥] Paul J. Saines,[*,¶] Paolo Barone,[§] Alessandro Stroppa,[*,∥] and Jaejun Yu[*,‡]

†*Korea Institute for Advanced Study, Seoul 02455, Republic of Korea*

‡*Center for Theoretical Physics, Department of Physics and Astronomy, Seoul National University, Seoul 08826, Republic of Korea*

¶*School of Chemistry and Forensic Science, University of Kent, Canterbury CT2 7NH, United Kingdom*

§*Consiglio Nazionale delle Ricerche, Institute for Superconducting and Innovative Materials and Devices (CNR-SPIN), Area della Ricerca di Tor Vergata, Via del Fosso del Cavaliere 100, 00133 Rome, Italy*

∥*Consiglio Nazionale delle Ricerche, Institute for Superconducting and Innovative Materials and Devices (CNR-SPIN), c/o Department of Physical and Chemical Sciences, University of L'Aquila, Via Vetoio I-67100 Coppito, L'Aquila, Italy*

⊥*Contributed equally to this work*

E-mail: P.Saines@kent.ac.uk; alessandro.stroppa@spin.cnr.it; jyu@snu.ac.kr

S1. Additional Sample Preparation Details

Solvents were purchased from Fisher, degassed, and stored under argon before use; Et_2O was dried by reflux over potassium and stored a potassium mirror. $CrCl_3.6H_2O$ and $CrCl_2$ were purchased from Aldrich, guanidinium carbonate and formic acid were purchased from Acros, sulfuric acid was purchased from Fisher and chromium metal was purchased from Hopkin and Williams Ltd.; all were used as obtained without further purification.

For the synthesis method use to produce single crystals of **1-Cr** $[C(NH_2)_3](HCOO)$ was made by suspending $[C(NH_2)_3]_2CO_3$ (5.00 g, 27.8 mmol) in isopropanol (20 cm^3). A solution of H_2COO (2.5 cm^3, 66 mmol) in isopropanol (10 cm^3) was added dropwise with stirring over the course of 20 minutes, resulting in significant effervescence and dissolution of the solid. The reaction mixture was stirred overnight, and the solvent removed in vacuo to give a colorless liquid. This was triturated with 30 cm^3 diethyl ether to give a colorless solid. The liquor was removed by canula filtration and the solid dried in vacuo to give crude guanidinium formate as a colorless solid (5.45 g, 52 mmol, 93% yield) which was used without further purification.

The solution of $[C(NH_2)_3](HCOO)$ used for preparation of the bulk sample was made by suspending $[C(NH_2)_3](CO_3)$ (1.81 g, 20 mmol) in H_2O (10 cm^3) with H_2COO (1.88 cm^3, 50 mmol) then added dropwise. This induced rapid effervescence, which ceased after the reaction was stirred for a further hour, leading to the formation of a colorless solution. This solution was heated to boiling briefly to remove CO_2 and then allowed to cool to ambient temperature.

S2. Single Crystal Diffraction Measurements and Analysis

SCXRD studies utilized Cu-Kα (λ = 1.54184 Å) radiation with single crystals mounted on MiTeGen microloops with temperature control via an Oxford Cryosystems Cryostream. Unit cell determination, data reduction and absorption corrections were performed using

CrysAlisPro 171.38.43.[S1] The structures were solved with the SHELXT structure solution program[S2] via intrinsic phasing controlled via the Olex2 GUI[S3] and subsequently refined with the SHELXL refinement package using least squares minimisation[S4] (See Table S1 for crystallographic details). Non-hydrogen atoms were refined anisotropically, and hydrogen atoms were included using a riding model with displacement parameters constrained to be 1.2 or 1.5 times that of the N or C to which they are attached.

The structure obtained at 100 K is compared with the theoretically predicted structure used for the DFT calculations. The lattice constants used for the DFT calculations deviate from the experimental lattice constants by −0.19 %, −0.79 %, and −1.56 % for a, b, and c axes, respectively. For the internal coordinates of atoms, the quantitative comparison was performed by using COMPSTRU of Bilbao Crystallographic Server.[S5] In terms of the quantities defined therein, the differences are represented by the degree of lattice distortion $S = 0.0060$, the maximum distance $d_{\mathrm{max.}} = 0.2705$ Å, the arithmetic mean of the distance $d_{\mathrm{av.}} = 0.1171$ Å, and the measure of similarity $\Delta = 0.038$. From these values, we conclude that the two structures are close enough to justify our DFT description.

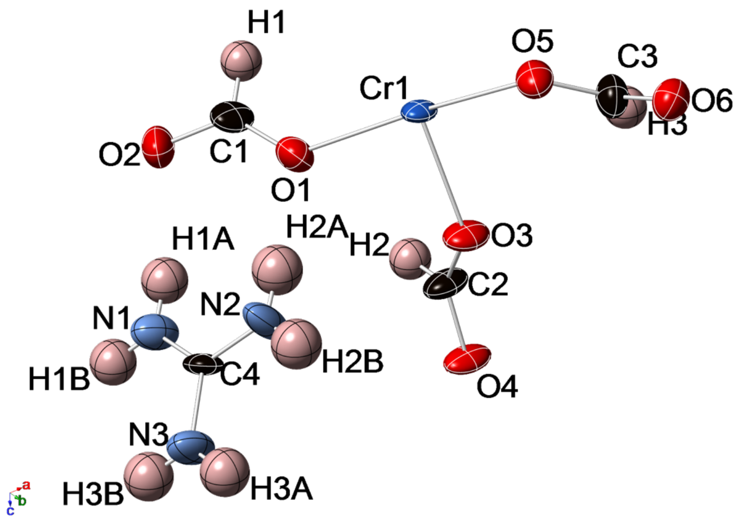

Figure S1: Asymmetric unit of **1-Cr** at 300 K with atoms shown as thermal ellipsoids with 40 % probability. Atoms are colored as in Fig. 1.

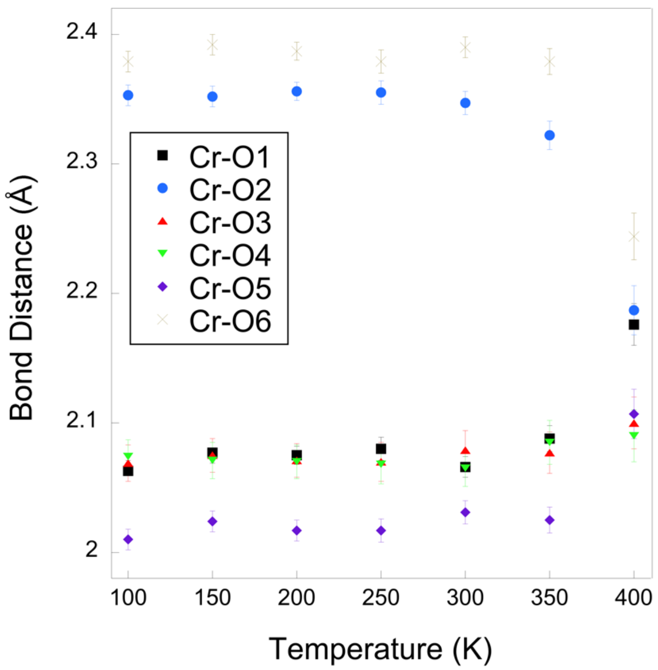

Figure S2: Plot of the Cr-O bond distances in **1-Cr** as a function of temperature.

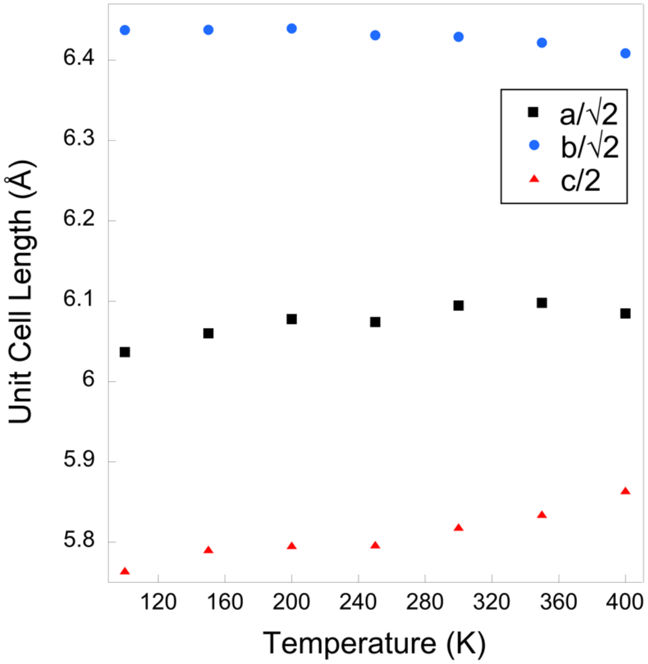

Figure S3: Plot of lattice parameters versus temperature for **1-Cr**.

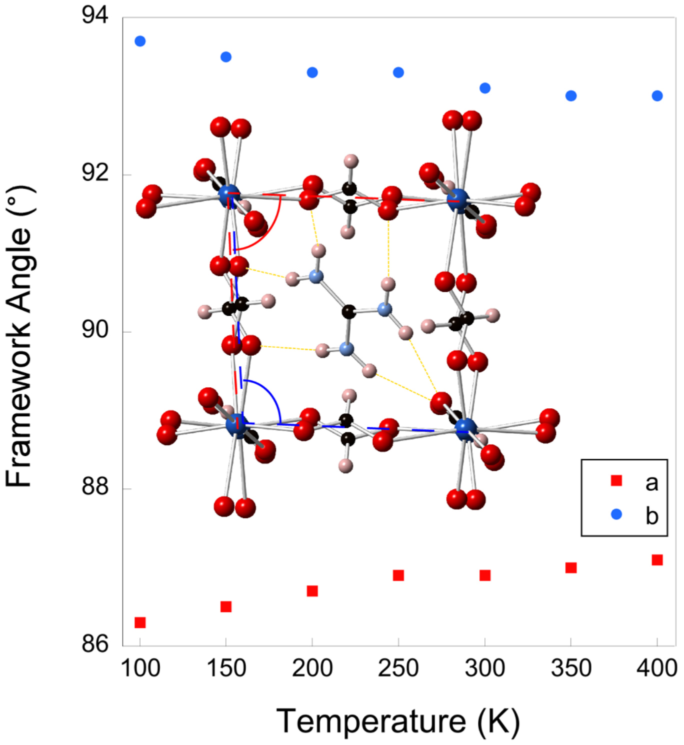

Figure S4: Depiction of the evolution of the framework angles along the a and b axis for **1-Cr**. Insert indicates the orientation of these framework angles in the structure, atoms are shown in the same color in Fig. 1. Hydrogen bonds are shown between the $[C(NH_2)_3]^+$ cation and the anionic $[Cr(HCOO)_3]^-$ framework as dashed gold lines; each hydrogen of the $[C(NH_2)_3]^+$ cation forms a single hydrogen bond with an oxygen atom from a formate ligand with D...A distances of 2.933(19) to 3.019(13) Å.

Table S1: Crystallographic data for **1-Cr** determined using single crystal X-ray diffraction between 100-400 K. The single crystal was treated as a racemic twin.

Compound	100 K	150 K	200 K	250 K	300 K	350 K	400 K
Formula	$C_4H_9CrN_3O_6$	$C_4H_9CrN_3O_6$	$C_4H_9CrN_3O_6$	$C_4H_9CrN_3O_6$	$C_4H_9CrN_3O_6$	$C_4H_9CrN_3O_6$	$C_4H_9CrN_3O_6$
Formula Weight	247.14	247.14	247.14	247.14	247.14	247.14	247.14
Crystal System	Orthorhombic	Orthorhombic	Orthorhombic	Orthorhombic	Orthorhombic	Orthorhombic	Orthorhombic
Space Group	$Pna2_1$ (33)	$Pna2_1$ (33)	$Pna2_1$ (33)	$Pna2_1$ (33)	$Pna2_1$ (33)	$Pna2_1$ (33)	$Pna2_1$ (33)
a (Å)	8.537(2)	8.570(2)	8.5953(18)	8.590(3)	8.619(2)	8.624(2)	8.605(3)
b (Å)	9.104(2)	9.1043(19)	9.107(2)	9.095(3)	9.092(2)	9.082(3)	9.063(3)
c (Å)	11.529(3)	11.582(3)	11.592(3)	11.594(4)	11.638(3)	11.670(4)	11.729(4)
V (Å^3)	896.1(4)	903.7(4)	907.4(4)	905.7(5)	911.9(4)	914.0(5)	914.8(6)
Z	4	4	4	4	4	4	4
ρ_{calc} (g cm^{-3})	1.832	1.816	1.809	1.812	1.800	1.796	1.795
μ (cm^{-1})	10.717	10.627	10.584	10.603	10.531	10.507	10.498
Average I/σ	19.5	17.2	18.9	17.4	16.7	15.9	11.9
Refl. Meas./Unique	1962/1180 [$R_{\mathrm{int}} = 0.0404$]	2000/1179 [$R_{\mathrm{int}} = 0.0422$]	1986/1171 [$R_{\mathrm{int}} = 0.0459$]	1974/1169 [$R_{\mathrm{int}} = 0.0538$]	1996/1181 [$R_{\mathrm{int}} = 0.0558$]	1998/1183 [$R_{\mathrm{int}} = 0.0559$]	2001/1179 [$R_{\mathrm{int}} = 0.0682$]
Parameters Refined	128	128	128	128	128	128	128
R_1, wR_2 ^{a} (all)	0.0914, 0.2315	0.0906, 0.2278	0.0778, 0.2077	0.0988, 0.2544	0.0863, 0.2307	0.1117, 0.2568	0.1183, 0.2600
R_1, wR_2 ^{a} (obs)	0.0876, 0.2409	0.0852, 0.2176	0.0723, 0.1975	0.0917, 0.2369	0.0764, 0.2112	0.0959, 0.2305	0.0807, 0.2109
GOF	1.093	1.074	1.048	1.087	1.047	1.052	1.013

^{a} $w = 1/[\sigma^2(F_o^2) + (aP)^2 + bP]$ and $P = (\max(F_o^2, 0) + 2F_c^2)/3$; $R_1 = \Sigma||F_o| - |F_c||/\Sigma|F_o|$ and $wR_2 = \sqrt{[(F_o^2 - F_c^2)^2/\Sigma w(F_o^2)^2]}$

Table S2: Cation coordination bond angles in ° at 300 K for **1-Cr**.

O1-Cr1-O6	81.3(3)	O1-Cr1-O3	89.6(5)	O4-Cr1-O5	91.0(5)
O5-Cr1-O6	85.3(3)	O2-Cr1-O3	89.8(5)	O2-Cr1-O5	107.0(3)
O1-Cr1-O2	86.4(4)	O4-Cr1-O6	90.6(4)	O1-Cr1-O5	166.5(3)
O3-Cr1-O5	88.9(4)	O3-Cr1-O6	90.7(3)	O2-Cr1-O6	167.7(3)
O2-Cr1-O4	88.9(4)	O1-Cr1-O4	90.8(4)	O3-Cr1-O4	178.7(4)

S3. Symmetry Analysis of Magnetic Ground State

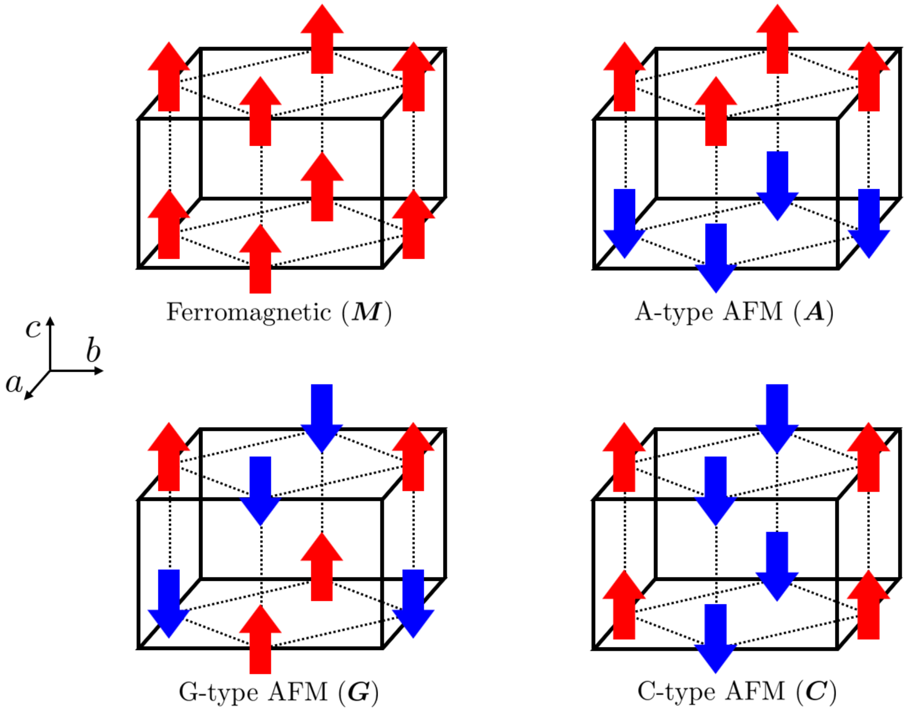

Figure S5: The spin configurations corresponding to the ferromagnetic ($\boldsymbol{M}$), G-type AFM ($\boldsymbol{G}$), A-type AFM ($\boldsymbol{A}$), and C-type AFM ($\boldsymbol{C}$) orders, respectively. Upward red and downward blue arrows mean the spin-up and spin-down, respectively. The dotted lines represent the neighboring spin pairs and the solid line box is a half of the unit cell of **1-Cr/Cu** by {**a**, **b**, **c**/2}. The figure shows the c-component case of each order, such as A_c. The $b(c)$-component can be obtained by setting the $b(c)$-axis as the spin-axis.

The space group of the **1-Cr** crystal is $Pna2_1$, comprising four crystalline symmetry operations: a twofold rotation 2_z about the z axis followed by a translation of half the c lattice constant, and two mirror operations m_x, m_y whose planes are perpendicular to the a and b axes respectively. One can define four magnetic order parameters per cell:

$$\begin{aligned} \boldsymbol{M} &= \boldsymbol{S}_1 + \boldsymbol{S}_2 + \boldsymbol{S}_3 + \boldsymbol{S}_4 \\ \boldsymbol{G} &= \boldsymbol{S}_1 + \boldsymbol{S}_2 - \boldsymbol{S}_3 - \boldsymbol{S}_4 \\ \boldsymbol{A} &= \boldsymbol{S}_1 - \boldsymbol{S}_2 + \boldsymbol{S}_3 - \boldsymbol{S}_4 \\ \boldsymbol{C} &= \boldsymbol{S}_1 - \boldsymbol{S}_2 - \boldsymbol{S}_3 + \boldsymbol{S}_4 \end{aligned} \tag{S1}$$

where the positions of magnetic (Cr) ions are given by the $4a$ Wyckoff positions of the $Pna2_1$ space group:

$$\begin{aligned}
Cr1 &: x, y, z \\
Cr2 &: -x+1/2, y+1/2, z+1/2 \\
Cr3 &: x+1/2, -y+1/2, z \\
Cr4 &: -x, -y, z+1/2
\end{aligned} \tag{S2}$$

Starting from the paramagnetic space group $Pna2_11'$, the transformation rules for the order parameters introduced before in Eq. (S1) can be readily obtained and are given in Table S3. Using these transformation rules, it is straightforward to identify allowed couplings between

Table S3: Transformation rules of magnetic order parameters and corresponding magnetic irrep (isotropy subgroups also given in brackets).

	1	2_c	m_a	m_b	1'	mIR	
M_a	+1	-1	+1	-1	-1	mΓ_3	$(Pna'2'_1)$
M_b	+1	-1	-1	+1	-1	mΓ_4	$(Pn'a2'_1)$
M_c	+1	+1	-1	-1	-1	mΓ_2	$(Pn'a'2_1)$
G_a	+1	+1	+1	+1	-1	mΓ_1	$(Pna2_1)$
G_b	+1	+1	-1	-1	-1	mΓ_2	$(Pn'a'2_1)$
G_c	+1	-1	-1	+1	-1	mΓ_4	$(Pn'a2'_1)$
A_a	+1	+1	-1	-1	-1	mΓ_2	$(Pn'a'2_1)$
A_b	+1	+1	+1	+1	-1	mΓ_1	$(Pna2_1)$
A_c	+1	-1	+1	-1	-1	mΓ_3	$(Pna'2'_1)$
C_a	+1	-1	-1	+1	-1	mΓ_4	$(Pn'a2'_1)$
C_b	+1	-1	+1	-1	-1	mΓ_3	$(Pna'2'_1)$
C_c	+1	+1	+1	+1	-1	mΓ_1	$(Pna2_1)$

different magnetic order parameters, as well as to tailor the possible allowed secondary order parameters generated by non-collinear arrangements of canted magnetic moments. In general, four magnetic space groups can entail the magnetic configuration of the $Pna2_1$ structure, i.e., $Pna2_1, Pn'a'2_1, Pna'2'_1$ and $Pn'a2'_1$. As consistent with the previous studies,[S6,S7] our DFT($+U+J$) calculations confirm that the A-type AFM always displays the lowest energy and thus ruling out the magnetic space group $Pn'a2'_1$. Assuming that the primary order

parameter is $\boldsymbol{A}$, the following three situations are compatible with the symmetry properties:

$$\begin{aligned} Pna2_1 &: \{A_b; G_a, C_c\} \\ Pn'a'2_1 &: \{A_a; G_b, M_c\} \\ Pna'2'_1 &: \{A_c; C_b, M_a\} \end{aligned} \tag{S3}$$

two of which are compatible with weak ferromagnetic components (parallel to a and c axes). DFT calculations for **1-Cr** show that A_a corresponds to the most stable magnetic configuration.

S4. Spin Model

Magnetic properties are modeled via an isotropic 3D Heisenberg model for classical spins (with $S = 2$):

$$H_{iso} = \frac{1}{2}\sum_{ij} J_{ij}\, \boldsymbol{S}_i \cdot \boldsymbol{S}_j \tag{S4}$$

Two different (bond-anisotropy) exchange interactions are considered: $J_c > 0$, denoting the AFM exchange coupling along the c axis, and $J_{ab} < 0$, denoting the FM interaction within ab planes. The isotropic spin model is supplemented by a site-dependent single-ion anisotropy (SIA) defined as[S7]

$$H_{sia} = E\sum_{i} \boldsymbol{S}_i \cdot \boldsymbol{R}(\theta_{\mathrm{t},i})\mathcal{A}_i\boldsymbol{R}(\theta_{\mathrm{t},i})^{-1} \cdot \boldsymbol{S}_i \tag{S5}$$

Here the local SIA tensor, accounting for the JT-induced orbital order, is given in the local reference frame of distorted CrX_6 octahedra by

$$\mathcal{A}_i = \begin{pmatrix} (-1)^{i_x+i_y}E & 0 & 0 \\ 0 & (-1)^{i_x+i_y+1}E & 0 \\ 0 & 0 & D \end{pmatrix} \tag{S6}$$

and $\boldsymbol{R}(\theta_{\mathrm{t},i})$ is a rotation matrix that accounts for the effect of tilting rotations on SIA:

$$\boldsymbol{R}(\theta_{\mathrm{t},i}) = \begin{pmatrix} \frac{1}{2}(\cos\theta_{\mathrm{t},i}+1) & \frac{1}{2}(1-\cos\theta_{\mathrm{t},i}) & \frac{1}{\sqrt{2}}\sin\theta_{\mathrm{t},i} \\ \frac{1}{2}(1-\cos\theta_{\mathrm{t},i}) & \frac{1}{2}(\cos\theta_{\mathrm{t},i}+1) & -\frac{1}{\sqrt{2}}\sin\theta_{\mathrm{t},i} \\ -\frac{1}{\sqrt{2}}\sin\theta_{\mathrm{t},i} & \frac{1}{\sqrt{2}}\sin\theta_{\mathrm{t},i} & \cos\theta_{\mathrm{t},i} \end{pmatrix} \tag{S7}$$

with $\theta_{\mathrm{t},i} = (-1)^{i_x+i_y+i_z}\theta_{\mathrm{t}}$, and $\theta_{\mathrm{t},i}$ the structural tilting angle of $\mathrm{CrX_6}$ octahedra. Note that we are using the different notation θ_{t} from Ref. S7 (α) for the tilting angle for consistency with the main text. The global cartesian reference frame has the spin quantization axis z parallel to the crystal axis c, while the x, y axes are rotated by 45° with respect to the a, b axes.[S7]

Assuming an A-AFM configuration aligned along the a-axis, we modeled the secondary ferromagnetic and AFM order parameters. The mean-field energy of the model can be evaluated assuming the following expressions for the local magnetic moments:

$$
\begin{aligned}
\boldsymbol{S}_1 &= (\cos\epsilon\cos(\phi+\delta), \cos\epsilon\sin(\phi+\delta), -\sin\epsilon) \\
\boldsymbol{S}_2 &= (\cos\epsilon\cos(\phi-\pi+\delta), \cos\epsilon\sin(\phi-\pi+\delta), -\sin\epsilon) \\
\boldsymbol{S}_3 &= (\cos\epsilon\cos(\phi-\delta), \cos\epsilon\sin(\phi-\delta), -\sin\epsilon) \\
\boldsymbol{S}_4 &= (\cos\epsilon\cos(\phi-\pi-\delta), \cos\epsilon\sin(\phi-\pi-\delta), -\sin\epsilon) \qquad \text{(S8)}
\end{aligned}
$$

where $\phi = \pi/4$ to enforce the primary order parameter A_a, while δ, ϵ account for the canting producing weak G_b, M_c components, respectively. The mean-field energy reads:

$$
\begin{aligned}
E(\delta,\epsilon) &= -J_c\cos 2\epsilon - 2|J_{ab}|\left[1+\cos^2\epsilon(\cos 2\delta - 1)\right] + D\cos^2\theta_{\mathrm{t}}\sin^2\epsilon - 2E\cos\theta_{\mathrm{t}}\cos^2\epsilon\sin 2\delta \\
&\quad + \frac{D}{2}\sin^2\theta_{\mathrm{t}}\cos^2\epsilon(1-\cos 2\delta) + \sin\theta_{\mathrm{t}}\sin 2\epsilon\left(D\cos\theta_{\mathrm{t}}\sin\delta + E\cos\delta\right) \qquad \text{(S9)}
\end{aligned}
$$

The canting angles can be determined by minimizing Eq. (S9) with respect to both δ and ϵ. Simple analytical formulas are obtained assuming $\delta = 0$ or $\epsilon = 0$, giving respectively (Note a corrected sign for ϵ to Ref. S7):

$$
\tan 2\epsilon = -\frac{2E\sin\theta_{\mathrm{t}}}{2J_c + D\cos^2\theta_{\mathrm{t}}} \qquad \text{(S10)}
$$

$$
\tan 2\delta = \frac{2E\cos\theta_{\mathrm{t}}}{2|J_{ab}| + D\sin^2\theta_{\mathrm{t}}} \qquad \text{(S11)}
$$

We notice that the ferromagnetic canting angle scales roughly as $1/J_c$, while the G-AFM angle $\delta \sim 1/J_{ab}$.

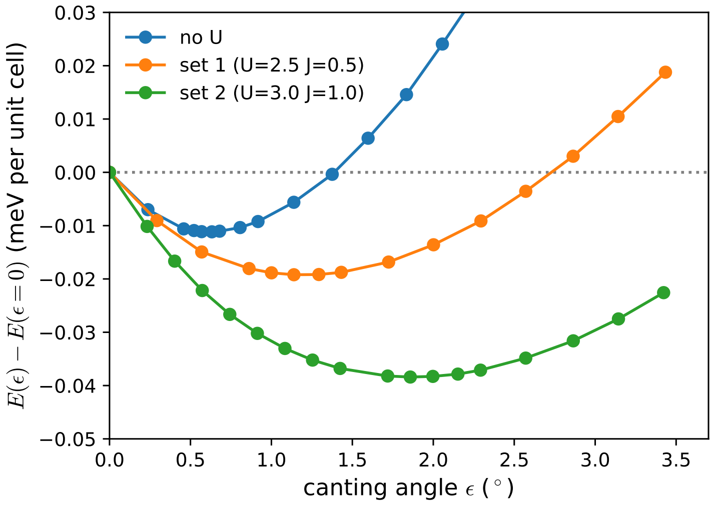

Figure S6: DFT total energies of **1-Cr** depending on the canting angle ϵ in ac-plane. Without the U and J corrections (no U), the total energy is minimum at $\epsilon = 0.63°$. With the DFT+$U + J$ correction, the minima are at $\epsilon = 1.19°$ and $\epsilon = 1.86°$ for the parameter set 1 (2.5,0.5) and set 2 (3,1), respectively. In a similar way, the optimal canting angle of the G_b order is $\delta = 2.01°$ (no U).

Model parameters can be estimated by total-energy mapping of DFT computations, as done in the Supplementary Information of Ref. S7 (Note that the method therein is formulated as 'per unit cell' values). In addition, constrained spin calculations were performed to obtain the optimal canting angle ϵ as shown in Fig. S6. Two sets of parameters have been used, as listed in Table 1 of the main text, corresponding to DFT+U results with fixed $U - J = 2$ eV but with $(U, J) = (2.5, 0.5)$ or $(U, J) = (3, 1)$. Since hybrid compounds containing Cr^{2+} ions are rare, there is no available reference of the U parameter. Therefore, the choice of these parameters is arbitrary. Note that $J_c > |J_{ab}|$ implies that the G-AFM canting dominates over the weak ferromagnetic component because $\epsilon \sim 1/J_c$ and $\delta \sim 1/J_{ab}$.

We briefly comment on the possible role of the Dzyaloshinskii-Moriya interaction (DMI),[S8,S9] that in the previous work[S7] was shown to be incompatible with $\{A_a; M_c\}$ or $\{A_c; M_a\}$ spin orderings. When we consider two Cr ions neighboring along c-direction with either one of those two spin orderings, the octahedra tilting implies that the DM vector is parallel to a-axis ($\boldsymbol{D} \parallel \hat{a}$). On the other hand, the cross-product of two spins is parallel to the b-axis

($\boldsymbol{S}_1 \times \boldsymbol{S}_2 \parallel \hat{b}$). Since the DMI term in the Hamiltonian is usually described in the form of $\boldsymbol{D} \cdot (\boldsymbol{S}_1 \times \boldsymbol{S}_2) = 0$, it can not affect the spin canting. However, this is not the case in the presence of the G_b or C_b orders, which are not considered in the previous work.[S7] Let us first consider the $\{A_a; G_b, M_c\}$ case ($Pn'a'2_1$). As a first-order expansion, we can write $\boldsymbol{S}_1 = S(1, \delta, \epsilon)$ and $\boldsymbol{S}_2 = S(-1, -\delta, \epsilon)$, which give $\boldsymbol{S}_1 \times \boldsymbol{S}_2 = S^2(2\delta\epsilon, -2\epsilon, 0)$. When we write $\boldsymbol{D} = (D, 0, 0)$, the DMI interaction is $\boldsymbol{D} \cdot (\boldsymbol{S}_1 \times \boldsymbol{S}_2) = 2DS^2\delta\epsilon$, which is no longer zero. Hence, we can not strictly rule out the DMI as an origin of the spin canting. Nevertheless, we can ignore the DMI in practice for the **1-Cr** where we adopted $Pn'a'2_1 : \{A_a; G_b, M_c\}$ order because the DMI energy $2DS^2\delta\epsilon$ is a second-order term in the small canting angles. On the other hand, in the case of $\{A_c; C_b, M_a\}$ ($Pna'2'_1$), we can write $\boldsymbol{S}_1 = S(\epsilon, \delta, 1)$ and $\boldsymbol{S}_2 = S(\epsilon, \delta, -1)$ (in this case, ϵ for M_a and δ for C_b). It leads to $\boldsymbol{S}_1 \times \boldsymbol{S}_2 = S^2(2\delta\epsilon, -2\epsilon, 0)$ and $\boldsymbol{D} \cdot (\boldsymbol{S}_1 \times \boldsymbol{S}_2) = -2DS^2\delta$. The DMI is linear to δ, which does not induce a net WFM moment. Therefore, the original argument of dismissing the DMI is still valid for $Pna'2'_1$, which corresponds to the **1-Cu**.

S5. Monte Carlo Results

Monte Carlo simulations have been performed on a L×L×L supercell, with $L = 10, 20$ or 24, comprising 4000, 32000, or 55296 spins, respectively. A standard Metropolis algorithm has been used, with 10^5 MC steps for equilibration and 10^5 MC steps for averaging.

The transition temperature can be estimated from the peak appearing in the specific heat per unit cell evaluated as

$$C_v = \frac{k_B \beta^2}{N_s} \left[\langle E^2 \rangle - \langle E \rangle^2 \right] \tag{S12}$$

where k_B is the Boltzmann constant, $\beta = 1/k_B T$, E the energy and N_s the number of spins in the simulation cell, while $\langle ... \rangle$ indicates statistical averages. One can similarly define the

(generalized) magnetic susceptibilities as

$$\chi^M = \beta N_s \left[\langle M^2\rangle - \langle M\rangle^2\right]$$
$$\chi^L = \beta N_s \left[\langle L^2\rangle - \langle L\rangle^2\right] \tag{S13}$$

where M is the ferromagnetic order parameter $L = A, C, G$ denotes the antiferromagnetic order parameter (all magnetic order parameters are defined per magnetic ion in MC calculations). A singularity in the generalized magnetic susceptibility allows identifying the primary magnetic order parameter.

Given the magnetic anisotropy, the full magnetic susceptibility tensor has also been evaluated, each component being defined accordingly as:

$$\chi^M_{\alpha\beta} = \beta N_s \left[\langle M_\alpha M_\beta\rangle - \langle M_\alpha\rangle\langle M_\beta\rangle\right] \tag{S14}$$

The "powder average" of the magnetic susceptibility is simply defined as $\chi^M_{av} = (\sum_\alpha \chi^M_{\alpha\alpha})/3$.

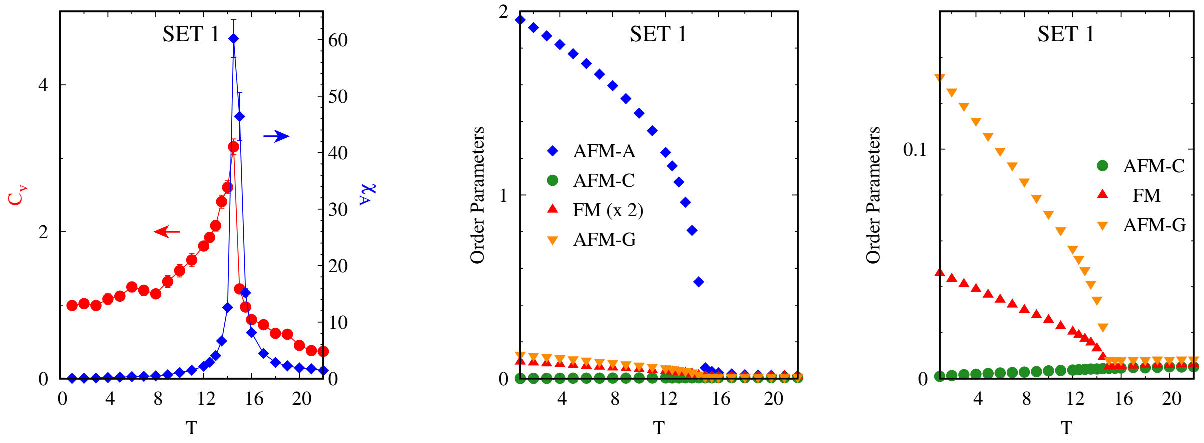

Figure S7: **Monte Carlo simulations (set 1).** Left panel: specific heat and A-type AFM susceptibility per unit cell as a function of temperature, both displaying a peak at temperatures within 14 and 15 K. Central panel: evolution with temperature of the A-type AFM order parameter alongside secondary order parameters. Note that the unit of susceptibility here is $\beta = 1/k_BT$ in meV, as a result of dimensionless spin in the simulation. Right panel: zoomed view of secondary order parameters, highlighting the small G-type AFM and weak FM components developing at the critical temperature. Results shown for $L = 24$.

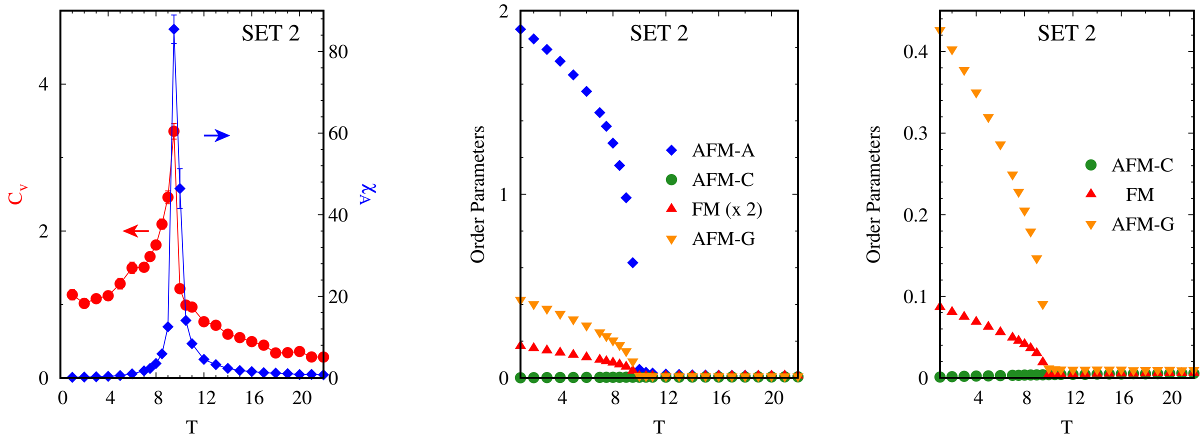

Figure S8: **Monte Carlo simulations (set 2).** Left panel: specific heat and A-type AFM susceptibility per unit cell as a function of temperature, both displaying a peak at temperatures within 9 and 10 K. Central panel: evolution with temperature of the A-type AFM order parameter alongside secondary order parameters. Right panel: zoomed view of secondary order parameters, highlighting the small G-type AFM and weak FM components developing at the critical temperature. Results shown for $L = 24$.

Results of MC calculations for $L = 24$ are summarized in Figs. S7 and S8, showing specific heat, primary order parameter A and associated susceptibility as well as secondary order parameters. The estimated critical temperature is $\sim$ 14.5 K for set 1 and $\sim$ 9.5 K for set 2. In both parameter sets, the dominant secondary order parameter appears to be G_b: the WFM component develops in both cases at the transition temperature, but it is one order of magnitude smaller than the primary AFM-A order parameter when using parameter set 1. In Fig. S9, the powder-averaged magnetic susceptibility is also shown for parameter set 2: despite the curve is still quite noisy, a qualitative trend can already be seen, showing a hump followed by a sizeable decrease as the temperature is lowered across the transition temperature T_c. The square of the magnetic moment is $\mu_B^2 S^2$ in the classical approach, whereas $\mu_B^2 S(S+1)$ in the quantum mechanics. When the χ_{av} is converted to the physical unit (emu mol^{-1} Oe^{-1}), this results in a difference of 1.5 times between classical and quantum cases for $S = 2$. In Fig. 3 (a) of the main text, the quantum mechanically converted values are used. Note that the previous study[S7] which ignored the U and J corrections predicted the critical temperature as about 40 K.

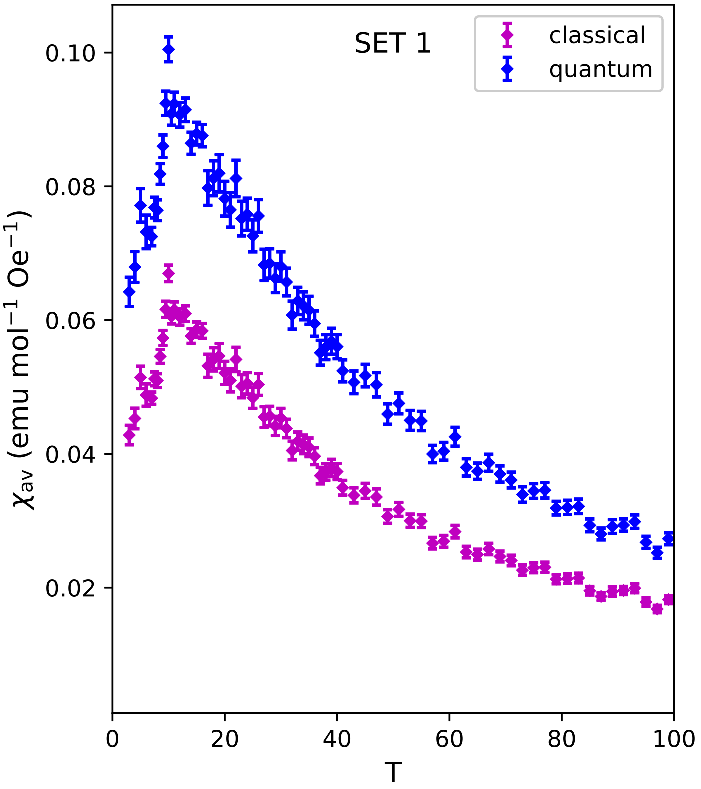

Figure S9: **Monte Carlo simulations (set 2).** Powder average of the magnetic susceptibility converted to a physical unit, showing a hump around the transition temperature. Results shown for $L = 24$. The labels mean in which way the unit is converted.

S6. DFT Parameter Dependence of Magnetic Moments

The calculated magnetic moments of the **1-Cr/Cu** can depend on the DFT parameters, as shown for the spin magnetic moment of **1-Cr** in the previous section. Table S4 shows the net spin and orbital weak ferromagnetic moments per unit cell (4 f.u.) and the magnitude of the orbital magnetic moment of a single Cr/Cu ion in the **1-Cr/Cu**. One can see that the orbital magnetic moments are enhanced by the inclusion of the $+U + J$ correction.

S7. Matrix Representations of the Spin-orbit Coupling

To express the SOC Hamiltonian $H_{\mathrm{SOC}} = \zeta \mathbf{S} \cdot \mathbf{L}$, let us denote the local coordinate unit vectors for spin operator as $(\mathbf{x}', \mathbf{y}', \mathbf{z}')$, and those for orbital angular momentum as $(\mathbf{x}, \mathbf{y}, \mathbf{z})$. H_{SOC}

Table S4: DFT parameter dependence of the calculated magnetic moments. Units are Bohr magneton μ_B. $|M_{\mathrm{orb}}|$ is the magnitude of orbital magnetic moment of single Cr/Cu ion.

	(U, J) (eV)	spin WFM comp. per unit cell	orbital WFM comp. per unit cell	$\lvert M_{\mathrm{orb}}\rvert$
**1-Cr**	$(0, 0)$	0.172	-0.024	0.031
**1-Cr**	$(2.5, 0.5)$	0.327	-0.031	0.035
**1-Cr**	$(3.0, 1.0)$	0.514	-0.032	0.035
**1-Cu**	$(0, 0)$	0.043	0.070	0.055
**1-Cu**	$(4.5, 0.5)$	0.054	0.096	0.077
**1-Cu**	$(5.0, 1.0)$	0.041	0.092	0.075

is parametrized by polar angle θ and azimuthal angle ϕ which describes the relative rotation of the spin coordinates, or simply the spin direction, with respect to the orbital coordinates, i.e., $s_x = \sin\theta\cos\phi$, $s_y = \sin\theta\sin\phi$, and $s_z = \cos\theta$. We rotate the primed coordinates with respect to the unprimed coordinates. Then the primed coordinate unit vectors are $\mathbf{x}' = \cos\theta\cos\phi\mathbf{x} + \cos\theta\sin\phi\mathbf{y} - \sin\theta\mathbf{z}$, $\mathbf{y}' = -\sin\phi\mathbf{x} + \cos\phi\mathbf{y}$, and $\mathbf{z}' = \sin\theta\cos\phi\mathbf{x} + \sin\theta\sin\phi\mathbf{y} + \cos\theta\mathbf{z}$. The SOC Hamiltonian is written as[S10]

$$\begin{aligned} H_{\mathrm{SOC}} = \zeta\mathbf{S}\cdot\mathbf{L} = &\frac{\zeta}{2}\begin{pmatrix} \sin\theta\cos\phi & \cos\theta\cos\phi + i\sin\phi \\ \cos\theta\cos\phi - i\sin\phi & -\sin\theta\cos\phi \end{pmatrix} L_x \\ &+ \frac{\zeta}{2}\begin{pmatrix} \sin\theta\sin\phi & \cos\theta\sin\phi - i\cos\phi \\ \cos\theta\sin\phi + i\cos\phi & -\sin\theta\sin\phi \end{pmatrix} L_y \\ &+ \frac{\zeta}{2}\begin{pmatrix} \cos\theta & -\sin\theta \\ -\sin\theta & -\cos\theta \end{pmatrix} L_z. \end{aligned} \tag{S15}$$

The matrix representation for the orbital angular momentum operator is determined by the quantum mechanical relations for the angular momentum states $L_z\,|l,m\rangle = m\,|l,m\rangle$ and $L_\pm\,|l,m\rangle = \sqrt{(l \mp m)(l \pm m + 1)}\,|l, m \pm 1\rangle$. We take d-orbitals ($l = 2$) in the real spherical harmonics form as a basis: $|yz\rangle = (i/\sqrt{2})(|2,-1\rangle + |2,+1\rangle)$, $|zx\rangle = (1/\sqrt{2})(|2,-1\rangle - |2,+1\rangle)$, $|xy\rangle = (i/\sqrt{2})(|2,-2\rangle - |2,2\rangle)$, $|x^2-y^2\rangle = (1/\sqrt{2})(|2,-2\rangle + |2,+2\rangle)$, and $|z^2\rangle = |2,0\rangle$. Having the order of basis in $\{d_{yz}, d_{zx}, d_{xy}, d_{x^2-y^2}, d_{z^2}\}$, matrix representations of L_i's are as

follows in the atomic units.

$$L_x^d = \begin{pmatrix} 0 & 0 & 0 & -i & -i\sqrt{3} \\ 0 & 0 & i & 0 & 0 \\ 0 & -i & 0 & 0 & 0 \\ i & 0 & 0 & 0 & 0 \\ i\sqrt{3} & 0 & 0 & 0 & 0 \end{pmatrix} \tag{S16}$$

$$L_y^d = \begin{pmatrix} 0 & 0 & -i & 0 & 0 \\ 0 & 0 & 0 & -i & i\sqrt{3} \\ i & 0 & 0 & 0 & 0 \\ 0 & i & 0 & 0 & 0 \\ 0 & -i\sqrt{3} & 0 & 0 & 0 \end{pmatrix} \tag{S17}$$

$$L_z^d = \begin{pmatrix} 0 & i & 0 & 0 & 0 \\ -i & 0 & 0 & 0 & 0 \\ 0 & 0 & 0 & 2i & 0 \\ 0 & 0 & -2i & 0 & 0 \\ 0 & 0 & 0 & 0 & 0 \end{pmatrix} \tag{S18}$$

The above orbital angular momentum operators can be redefined according to the JT effective Hamiltonian. The JT-rotated e_g-orbitals in Eq. (2) of the main text define the unitary matrix

$$U_0 = \begin{pmatrix} -\sin\left(\frac{\theta_{\mathrm{JT}}}{2}\right) & \cos\left(\frac{\theta_{\mathrm{JT}}}{2}\right) \\ \cos\left(\frac{\theta_{\mathrm{JT}}}{2}\right) & \sin\left(\frac{\theta_{\mathrm{JT}}}{2}\right) \end{pmatrix} \text{ and } U = \begin{pmatrix} \mathbf{I}_3 & \mathbf{0} \\ \mathbf{0} & U_0 \end{pmatrix}, \tag{S19}$$

where U is the unitary matrix for the whole d-orbitals. Consequently, the new orbital angular momentum operator matrices can be obtained by unitary rotation with this matrix, $(L_i^d)^{\mathrm{new}} = U^\dagger (L_i^d)^{\mathrm{old}} U$. The SOC in the JT distorted system can be expressed by using these

operators.

$$(L_x^d)^{\text{new}} = \begin{pmatrix} 0 & 0 & 0 & i\sin\left(\frac{\theta_{\text{JT}}}{2}\right) - i\sqrt{3}\cos\left(\frac{\theta_{\text{JT}}}{2}\right) & -i\cos\left(\frac{\theta_{\text{JT}}}{2}\right) - i\sqrt{3}\sin\left(\frac{\theta_{\text{JT}}}{2}\right) \\ & 0 & i & 0 & 0 \\ & & 0 & 0 & 0 \\ & & & 0 & 0 \\ h.c. & & & & 0 \end{pmatrix} \tag{S20}$$

$$(L_y^d)^{\text{new}} = \begin{pmatrix} 0 & 0 & -i & 0 & 0 \\ & 0 & 0 & i\sin\left(\frac{\theta_{\text{JT}}}{2}\right) + i\sqrt{3}\cos\left(\frac{\theta_{\text{JT}}}{2}\right) & -i\cos\left(\frac{\theta_{\text{JT}}}{2}\right) + i\sqrt{3}\sin\left(\frac{\theta_{\text{JT}}}{2}\right) \\ & & 0 & 0 & 0 \\ & & & 0 & 0 \\ h.c. & & & & 0 \end{pmatrix} \tag{S21}$$

$$(L_z^d)^{\text{new}} = \begin{pmatrix} 0 & i & 0 & 0 & 0 \\ & 0 & 0 & 0 & 0 \\ & & 0 & -2i\sin\left(\frac{\theta_{\text{JT}}}{2}\right) & 2i\cos\left(\frac{\theta_{\text{JT}}}{2}\right) \\ & & & 0 & 0 \\ h.c. & & & & 0 \end{pmatrix} \tag{S22}$$

In the following sections, we omit the superscripts 'd' and 'new' of L_i.

S8. Calculation of The Perturbed Orbital Angular Momentum

This section describes the detailed steps toward Eq. (3) in the main text. The unperturbed basis with the 'JT rotated' e_g-orbitals are $\{|d^0_{n\sigma}\rangle\}$ where $n = \{yz, zx, xy, -, +\}$ and $\sigma =\uparrow$

or $\downarrow$ spins. The first order corrected d-orbitals are

$$|d_\alpha\rangle = \left|d^0_\alpha\right\rangle + \sum_{\beta\neq\alpha} \frac{\left\langle d^0_\beta\middle|H_{SOC}\middle|d^0_\alpha\right\rangle}{E^0_\alpha - E^0_\beta} \left|d^0_\beta\right\rangle \tag{S23}$$

where the α and β are combined indices of orbital species and spin. The orbital angular momentum expectation value for a perturbed d-orbital $d_{n\uparrow}$ is

$$\begin{aligned}\langle d_{n\uparrow}|L_i|d_{n\uparrow}\rangle = &\left\langle d^0_{n\uparrow}\middle|L_i\middle|d^0_{n\uparrow}\right\rangle \\ &+ \sum_{m\neq n}\left[\tfrac{\left\langle d^0_{m\uparrow}\middle|H_{SOC}\middle|d^0_{n\uparrow}\right\rangle}{E^0_{n\uparrow}-E^0_{m\uparrow}}\left\langle d^0_{n\uparrow}\middle|L_i\middle|d^0_{m\uparrow}\right\rangle + c.c.\right] \\ &+ \sum_{\text{all } m}\left[\tfrac{\left\langle d^0_{m\downarrow}\middle|H_{SOC}\middle|d^0_{n\uparrow}\right\rangle}{E^0_{n\uparrow}-E^0_{m\downarrow}}\left\langle d^0_{n\uparrow}\middle|L_i\middle|d^0_{m\downarrow}\right\rangle + c.c.\right] \\ &+ O(\zeta^2).\end{aligned} \tag{S24}$$

Because $\left\langle d^0_{n\uparrow}\middle|L_i\middle|d^0_{n\uparrow}\right\rangle = \left\langle d^0_{n\uparrow}\middle|L_i\middle|d^0_{m\downarrow}\right\rangle = 0$, only the second term remains up to the first order in ζ. For the summation over the corresponding terms to Eq. (S24) of occupied orbitals, the following property can be exploited. If we sum up all the orbital angular momentum expectation values of the perturbed d-orbitals with up-spin,

$$\sum_n \langle d_{n\uparrow}|L_i|d_{n\uparrow}\rangle = \sum_n\sum_{m\neq n}\left[\tfrac{\left\langle d^0_{m\uparrow}\middle|H_{SOC}\middle|d^0_{n\uparrow}\right\rangle}{E^0_{n\uparrow}-E^0_{m\uparrow}}\left\langle d^0_{n\uparrow}\middle|L_i\middle|d^0_{m\uparrow}\right\rangle + c.c.\right] = 0 \tag{S25}$$

because each of the terms is canceled with the term whose n and m are exchanged. The same holds for down spin. It makes it easy to calculate the orbital angular momentum for d^4 and d^9. For the spin-up high spin configuration of the Cr^{2+} ion (d^4), occupied d-orbitals are $\{d_{yz\uparrow}, d_{zx\uparrow}, d_{xy\uparrow}, d_{-\uparrow}\}$. Therefore, $\langle L_i\rangle_{d^4} = -\langle d_{+\uparrow}|L_i|d_{+\uparrow}\rangle$ for d^4. For the Cu^{2+} ion (d^9), only $d_{+\downarrow}$ is unoccupied. For d^9, by using $\left\langle d^0_{m\uparrow}\middle|H_{SOC}\middle|d^0_{n\uparrow}\right\rangle = -\left\langle d^0_{m\downarrow}\middle|H_{SOC}\middle|d^0_{n\downarrow}\right\rangle$ from the opposite signs of the diagonal components of the spin matrix in Eq. (S15) and $\left\langle d^0_{n\uparrow}\middle|L_i\middle|d^0_{m\downarrow}\right\rangle = 0$,

$\langle L_i \rangle_{d^9} = -\langle d_{+\downarrow}|L_i|d_{+\downarrow}\rangle = -\langle L_i \rangle_{d^4}$. The perturbed $|d_+\rangle$ is

$$
\begin{aligned}
|d_+\rangle = \left|d_+^0\right\rangle &+ \left(\frac{\zeta}{2}\right) \frac{\sin\theta\cos\phi\left(-i\cos\left(\frac{\theta_{\mathrm{JT}}}{2}\right) - i\sqrt{3}\sin\left(\frac{\theta_{\mathrm{JT}}}{2}\right)\right)}{E_+^0 - E_{yz}^0} \left|d_{yz}^0\right\rangle \\
&+ \left(\frac{\zeta}{2}\right) \frac{\sin\theta\sin\phi\left(-i\cos\left(\frac{\theta_{\mathrm{JT}}}{2}\right) + i\sqrt{3}\sin\left(\frac{\theta_{\mathrm{JT}}}{2}\right)\right)}{E_+^0 - E_{zx}^0} \left|d_{zx}^0\right\rangle + \left(\frac{\zeta}{2}\right) \frac{2i\cos\theta\cos\left(\frac{\theta_{\mathrm{JT}}}{2}\right)}{E_+^0 - E_{xy}^0} \left|d_{xy}^0\right\rangle .
\end{aligned} \tag{S26}
$$

The t_{2g} orbital components are included in this expression. This is why the orbital angular momentum can appear. In the d^4 configuration,

$$
\begin{aligned}
\langle L_i \rangle_{d^4} &= -\langle d_+|L_i|d_+\rangle \\
&= -\left\langle d_+^0\middle|L_i\middle|d_+^0\right\rangle \\
&- \left(\frac{\zeta}{2}\right) \frac{\sin\theta\cos\phi\left(-i\cos\left(\frac{\theta_{\mathrm{JT}}}{2}\right) - i\sqrt{3}\sin\left(\frac{\theta_{\mathrm{JT}}}{2}\right)\right)}{E_+^0 - E_{yz}^0} \left\langle d_+^0\middle|L_i\middle|d_{yz}^0\right\rangle + c.c. \\
&- \left(\frac{\zeta}{2}\right) \frac{\sin\theta\sin\phi\left(-i\cos\left(\frac{\theta_{\mathrm{JT}}}{2}\right) + i\sqrt{3}\sin\left(\frac{\theta_{\mathrm{JT}}}{2}\right)\right)}{E_+^0 - E_{zx}^0} \left\langle d_+^0\middle|L_i\middle|d_{zx}^0\right\rangle + c.c. \\
&- \left(\frac{\zeta}{2}\right) \frac{2i\cos\theta\cos\left(\frac{\theta_{\mathrm{JT}}}{2}\right)}{E_+^0 - E_{xy}^0} \left\langle d_+^0\middle|L_i\middle|d_{xy}^0\right\rangle + c.c.
\end{aligned} \tag{S27}
$$

Matrix elements $\langle d_n^0|L_i|d_m^0\rangle$ are given by Eq. (S20)-(S22).

S9. Details in The Model Analysis for 1-Cr/Cu

In order to apply Eq. (3) in the main text to the orbital magnetic moments of **1-Cr/Cu**, we take one out of four Cr/Cu ions in a unit cell of **1-Cr/Cu**, say Cr/Cu1, as a reference to describe the system (See Fig. S10 (a-c)). If we know the local MO_6 structure and the local moment of Cr/Cu ion at one site, those of other sites are determined by the space group and magnetic group symmetry. The transformation rules of the magnetic moment with respect to Cr/Cu1 by each symmetry operation belonging to $Pna'2_1'$ and $Pn'a'2_1$, where the identity operator 1 corresponds to Cr/Cu1 site, are listed in Table 2 in the main text.

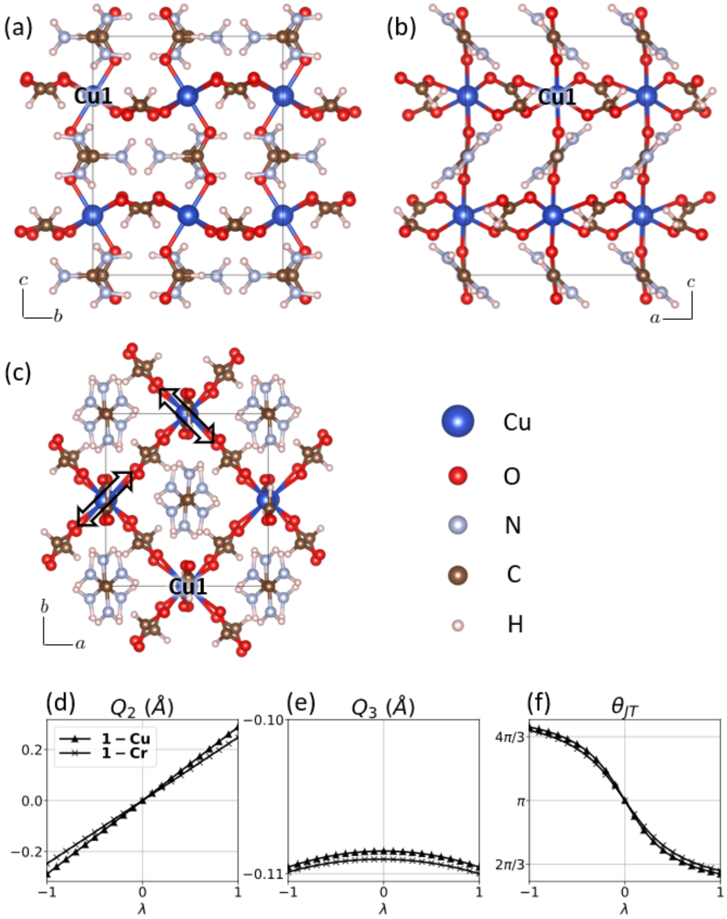

Figure S10: (a-c) $Pna2_1$ structure **1-Cu** ($\lambda = 1$). The reference Cu1 is labeled. In (c), elongated directions are drawn by double-arrows. JT mode amplitudes (d) Q_2 and (e) Q_3 of the reference Cu1 and Cr1 and their (f) JT phase are shown with respect to λ.

Let us consider the O_6 octahedron of the reference Cr/Cu1 ion. The local coordinate $(\mathbf{x}, \mathbf{y}, \mathbf{z})$ of the orbital magnetic moment is aligned to its O-M bond directions. The local coordinate system is determined by the following steps. First, put the octahedron in the way that the local coordinates $(\mathbf{x}, \mathbf{y}, \mathbf{z})$ are aligned with crystallographic $(\hat{\mathbf{a}}, \hat{\mathbf{b}}, \hat{\mathbf{c}})$ direction. Next, rotate the octahedron by $-\pi/4$ around the c axis, then by tilting angle $-\theta_t$ around

the a axis consecutively. As a result, $(\mathbf{x}, \mathbf{y}, \mathbf{z})$ can be written as follows.

$$\begin{aligned}
\mathbf{x} &= \tfrac{1}{\sqrt{2}}\hat{\mathbf{a}} - \tfrac{1}{\sqrt{2}}\cos\theta_{\mathrm{t}}\hat{\mathbf{b}} + \tfrac{1}{\sqrt{2}}\sin\theta_{\mathrm{t}}\hat{\mathbf{c}} \\
\mathbf{y} &= \tfrac{1}{\sqrt{2}}\hat{\mathbf{a}} + \tfrac{1}{\sqrt{2}}\cos\theta_{\mathrm{t}}\hat{\mathbf{b}} - \tfrac{1}{\sqrt{2}}\sin\theta_{\mathrm{t}}\hat{\mathbf{c}} \\
\mathbf{z} &= \sin\theta_{\mathrm{t}}\hat{\mathbf{b}} + \cos\theta_{\mathrm{t}}\hat{\mathbf{c}}
\end{aligned} \tag{S28}$$

The direction of the local spin magnetic moment of Cr/Cu1 is expressed in terms of the spherical coordinates θ_{spin} and ϕ_{spin} with respect to the local coordinate. If we ignore a small spin canting, the direction of spin is exactly c-direction in $Pna'2'_1$ magnetic group. It correspond to $\theta_{\mathrm{spin}} = \theta_{\mathrm{t}}$ and $\phi_{\mathrm{spin}} = -\frac{\pi}{4}$. For $Pn'a'2_1$, spin direction is a and corresponding angles are $\theta_{\mathrm{spin}} = \frac{\pi}{2}$ and $\phi_{\mathrm{spin}} = \frac{\pi}{4}$.

For the $Pna'2'_1$ symmetry, the total moment is four times the a-component of the moment of the reference Cr/Cu1. By using the above relations,

$$\langle L\rangle^{Pna'2'_1}_{\mathrm{total}} = 4\langle L\rangle_a = 4(\tfrac{1}{\sqrt{2}}\langle L_x\rangle + \tfrac{1}{\sqrt{2}}\langle L_y\rangle) \tag{S29}$$

Likewise, for the $Pn'a'2_1$ symmetry, the total moment is four times the c-component of the moment of the reference Cr/Cu1.

$$\langle L\rangle^{Pn'a'2_1}_{\mathrm{total}} = 4\langle L\rangle_c = 4(\tfrac{1}{\sqrt{2}}\sin\theta_{\mathrm{t}}\langle L_x\rangle - \tfrac{1}{\sqrt{2}}\sin\theta_{\mathrm{t}}\langle L_y\rangle + \cos\theta_{\mathrm{t}}\langle L_z\rangle) \tag{S30}$$

The inclusion of the small spin canting ($\epsilon < 1°$) would not significantly alter the predicted orbital magnetic moment. For example, the canting of 1° changes only about a factor of 0.0175 (1° in radians) of the results.

Let us consider the detailed aspect of the parametrization of the θ_{JT} by λ. In the **1-Cu**, as the λ increases from 0 to 1, JT mode Q_2 changes linearly from 0 to 0.288 as shown in Fig. S10 (d). Meanwhile, Q_3 changes very little, so it can be considered a constant (Fig. S10 (e)). $\lambda = 0$ and $\lambda = 1$ correspond to $\theta_{\mathrm{JT}} = \pi$ and $\theta_{\mathrm{JT}} = 1.934 \approx 0.616\pi$, respectively

(Fig. S10 (f)). This can be considered that $\tan(\pi - \theta_{\mathrm{JT}})$ is proportional to Q_2. This leads to the parametrization in the main text. The simplified value $\theta_{\mathrm{JT},\lambda=1} = 2\pi/3$ corresponds to $\left|d_-^0\right\rangle = -\left|x^2\right\rangle$ and $\left|d_+^0\right\rangle = -\left|y^2 - z^2\right\rangle$.

The parameters used to estimate A are as follows. We adopted SOC parameter $\zeta = 56.54$ meV for Cu and $\zeta = 27.52$ meV for Cr,[S11] $\Delta E = E_+^0 - E_{t_{2g}}^0 = 2.5$ eV for both of the **1-Cu** and **1-Cr**. ΔE is roughly chosen from the projected density of states obtained from the DFT calculation. The tilting angles of MO_6 octahedron extracted from the structures are $\theta_{\mathrm{t}} = 31.61°$ for **1-Cu** and $\theta_{\mathrm{t}} = 31.88°$ for **1-Cr**.

S10. The Second Order Energy Correction Term in The Perturbation Theory

In this section, we calculate the energy correction by the SOC in the perturbation approach, which gives rise to the MSIA. In the previous study,[S7] only the fixed JT phase and the same-spin contribution are considered for the MSIA. We improve the formulation by including the general JT phase and the opposite spin contribution. The first order energy correction $\langle d_{n\sigma}^0 | H_{\mathrm{SOC}} | d_{n\sigma}^0 \rangle$ vanishes because the diagonal components of L_i^d are 0. The lowest order energy correction is the second order correction term,

$$\Delta E_\alpha^2 = \sum_{\beta \neq \alpha} \frac{\left| \left\langle d_\beta^0 \middle| H_{SOC} \middle| d_\alpha^0 \right\rangle \right|^2}{E_\alpha^0 - E_\beta^0}. \tag{S31}$$

It is convenient to separate the H_{SOC} into the same-spin block and opposite-spin block.

$$H_{\mathrm{SOC},\uparrow\uparrow} = \frac{\zeta}{2}(\sin\theta\cos\phi L_x + \sin\theta\sin\phi L_y + \cos\theta L_x) = -H_{\mathrm{SOC},\downarrow\downarrow} \tag{S32}$$

and

$$H_{\mathrm{SOC},\uparrow\downarrow} = \frac{\zeta}{2}\left[(\cos\theta\cos\phi + i\sin\phi)L_x + (\cos\theta\sin\phi - i\cos\phi)L_y + (-\sin\theta)L_z\right] \tag{S33}$$

Similar to the orbital angular momentum case, the summation of the second-order correction to the energy with the non-degenerate assumption in the half-filling case with only up spins provides a shortcut for the calculation.

$$\begin{aligned}\sum_{\alpha\in\uparrow}\Delta E_\alpha^2 &= \sum_{\alpha\in\uparrow}\sum_{\beta\neq\alpha}\frac{\left|\left\langle d_\beta^0\middle|H_{SOC}\middle|d_\alpha^0\right\rangle\right|^2}{E_\alpha^0 - E_\beta^0}\\ &= \sum_n\sum_{m\neq n}\frac{\left|\left\langle d_{m\uparrow}^0\middle|H_{SOC,\uparrow\uparrow}\middle|d_{n\uparrow}^0\right\rangle\right|^2}{E_{n\uparrow}^0 - E_{m\uparrow}^0} + \sum_n\sum_m\frac{\left|\left\langle d_{m\downarrow}^0\middle|H_{SOC,\downarrow\uparrow}\middle|d_{n\uparrow}^0\right\rangle\right|^2}{E_{n\uparrow}^0 - E_{m\downarrow}^0}\end{aligned} \tag{S34}$$

The first term of the last line is the summation of the same-spin contribution to energy correction which vanishes. On the other hand, the second term is the summation of the opposite-spin contribution and is non-vanishing in general. It makes the difference between the expression of the energy correction of the d^4 spin configuration and that of the d^9.

The opposite-spin contributions from each orbital of the d^4 configuration are as follows

$$\begin{aligned}(\Delta E_{yz\uparrow}^2)_{\downarrow\uparrow} = \left(\tfrac{\zeta}{2}\right)^2\Big[&\frac{\sin^2\theta}{E_{yz\uparrow}^0 - E_{zx\downarrow}^0} + \frac{\cos^2\theta\sin^2\phi + \cos^2\phi}{E_{yz\uparrow}^0 - E_{xy\downarrow}^0}\\ &+ \frac{(-\sin(\theta_{\mathrm{JT}}/2) + \sqrt{3}\cos(\theta_{\mathrm{JT}}/2))^2(\cos^2\theta\cos^2\phi + \sin^2\phi)}{E_{yz\uparrow}^0 - E_{-\downarrow}^0}\\ &+ \frac{(\cos(\theta_{\mathrm{JT}}/2) + \sqrt{3}\sin(\theta_{\mathrm{JT}}/2))^2(\cos^2\theta\cos^2\phi + \sin^2\phi)}{E_{yz\uparrow}^0 - E_{+\downarrow}^0}\Big]\end{aligned} \tag{S35}$$

$$\begin{aligned}(\Delta E_{zx\uparrow}^2)_{\downarrow\uparrow} = \left(\tfrac{\zeta}{2}\right)^2\Big[&\frac{\sin^2\theta}{E_{zx\uparrow}^0 - E_{yz\downarrow}^0} + \frac{\cos^2\theta\cos^2\phi + \sin^2\phi}{E_{zx\uparrow}^0 - E_{xy\downarrow}^0}\\ &+ \frac{(\sin(\theta_{\mathrm{JT}}/2) + \sqrt{3}\cos(\theta_{\mathrm{JT}}/2))^2(\cos^2\theta\sin^2\phi + \cos^2\phi)}{E_{zx\uparrow}^0 - E_{-\downarrow}^0}\\ &+ \frac{(\cos(\theta_{\mathrm{JT}}/2) - \sqrt{3}\sin(\theta_{\mathrm{JT}}/2))^2(\cos^2\theta\sin^2\phi + \cos^2\phi)}{E_{zx\uparrow}^0 - E_{+\downarrow}^0}\Big]\end{aligned} \tag{S36}$$

$$(\Delta E^2_{xy\uparrow})_{\downarrow\uparrow} = \left(\tfrac{\zeta}{2}\right)^2 \Big[\frac{\cos^2\theta\sin^2\phi + \cos^2\phi}{E^0_{xy\uparrow} - E^0_{yz\downarrow}} + \frac{\cos^2\theta\cos^2\phi + \sin^2\phi}{E^0_{xy\uparrow} - E^0_{zx\downarrow}} + \frac{4\sin^2(\theta_{\rm JT}/2)\sin^2\theta}{E^0_{xy\uparrow} - E^0_{-\downarrow}} + \frac{4\cos^2(\theta_{\rm JT}/2)\sin^2\theta}{E^0_{xy\uparrow} - E^0_{+\downarrow}}\Big] \tag{S37}$$

$$(\Delta E^2_{-\uparrow})_{\downarrow\uparrow} = \left(\tfrac{\zeta}{2}\right)^2 \Big[\frac{(-\sin(\theta_{\rm JT}/2) + \sqrt{3}\cos(\theta_{\rm JT}/2))^2(\cos^2\theta\cos^2\phi + \sin^2\phi)}{E^0_{-\uparrow} - E^0_{yz\downarrow}} + \frac{(\sin(\theta_{\rm JT}/2) + \sqrt{3}\cos(\theta_{\rm JT}/2))^2(\cos^2\theta\sin^2\phi + \cos^2\phi)}{E^0_{-\uparrow} - E^0_{zx\downarrow}} + \frac{4\sin^2(\theta_{\rm JT}/2)\sin^2\theta}{E^0_{-\uparrow} - E^0_{xy\downarrow}}\Big] \tag{S38}$$

The spin direction-dependent terms including θ and ϕ can be rewritten in physically intuitive expressions,

$$\begin{aligned}
&\cos^2\theta\cos^2\phi + \sin^2\phi = 1 - \sin^2\theta\cos^2\phi = 1 - (\hat{\mathbf{s}}\cdot\mathbf{x})^2 \\
&\cos^2\theta\sin^2\phi + \cos^2\phi = 1 - \sin^2\theta\sin^2\phi = 1 - (\hat{\mathbf{s}}\cdot\mathbf{y})^2 \\
&\sin^2\theta = 1 - \cos^2\theta = 1 - (\hat{\mathbf{s}}\cdot\mathbf{z})^2,
\end{aligned} \tag{S39}$$

where $\hat{\mathbf{s}} = \mathbf{s}/|\mathbf{s}|$ is the unit vector indicating the spin direction. Meanwhile, it implies the existence of the spin direction independent contribution to the energy correction. The same-spin contribution to the correction is simply $(\Delta E^2_{d^4})_{\uparrow\uparrow} = -(\Delta E^2_{+\uparrow})_{\uparrow\uparrow}$ because the first term of the Eq. (S34) vanishes.

$$(\Delta E^2_{d^4})_{\uparrow\uparrow} = -\left(\tfrac{\zeta}{2}\right)^2 \Big[\frac{(\cos(\theta_{\rm JT}/2) + \sqrt{3}\sin(\theta_{\rm JT}/2))^2(\sin^2\theta\cos^2\phi)}{E^0_{+\uparrow} - E^0_{yz\uparrow}} + \frac{(\cos(\theta_{\rm JT}/2) - \sqrt{3}\sin(\theta_{\rm JT}/2))^2(\sin^2\theta\sin^2\phi)}{E^0_{+\uparrow} - E^0_{zx\uparrow}} + \frac{4\cos^2(\theta_{\rm JT}/2)\cos^2\theta}{E^0_{+\uparrow} - E^0_{xy\uparrow}}\Big] \tag{S40}$$

By summing up these terms, the spin direction-dependent part of the second order cor-

rection to the energy in d^4 configuration is

$$\begin{aligned}\Delta E^2_{d^4}(\mathbf{S}) = &\left(\tfrac{\zeta}{2}\right)^2(\hat{\mathbf{s}}\cdot\mathbf{x})^2\Big[\Big(\frac{-1}{E^0_{+\uparrow}-E^0_{yz\uparrow}}+\frac{1}{E^0_{+\downarrow}-E^0_{yz\uparrow}}\Big)(\cos(\theta_{\rm JT}/2)+\sqrt{3}\sin(\theta_{\rm JT}/2))^2\\
&+\Big(\frac{1}{E^0_{-\downarrow}-E^0_{yz\uparrow}}+\frac{1}{E^0_{yz\downarrow}-E^0_{-\uparrow}}\Big)(\sin(\theta_{\rm JT}/2)-\sqrt{3}\cos(\theta_{\rm JT}/2))^2+\frac{1}{E^0_{xy\downarrow}-E^0_{zx\uparrow}}+\frac{1}{E^0_{zx\downarrow}-E^0_{xy\uparrow}}\Big]\\
&+\left(\tfrac{\zeta}{2}\right)^2(\hat{\mathbf{s}}\cdot\mathbf{y})^2\Big[\Big(\frac{-1}{E^0_{+\uparrow}-E^0_{zx\uparrow}}+\frac{1}{E^0_{+\downarrow}-E^0_{zx\uparrow}}\Big)(\cos(\theta_{\rm JT}/2)-\sqrt{3}\sin(\theta_{\rm JT}/2))^2\\
&+\Big(\frac{1}{E^0_{-\downarrow}-E^0_{zx\uparrow}}+\frac{1}{E^0_{zx\downarrow}-E^0_{-\uparrow}}\Big)(\sin(\theta_{\rm JT}/2)+\sqrt{3}\cos(\theta_{\rm JT}/2))^2+\frac{1}{E^0_{xy\downarrow}-E^0_{yz\uparrow}}+\frac{1}{E^0_{yz\downarrow}-E^0_{xy\uparrow}}\Big]\\
&+\left(\tfrac{\zeta}{2}\right)^2(\hat{\mathbf{s}}\cdot\mathbf{z})^2\Big[\Big(\frac{-1}{E^0_{+\uparrow}-E^0_{xy\uparrow}}+\frac{1}{E^0_{+\downarrow}-E^0_{xy\uparrow}}\Big)4\cos^2(\theta_{\rm JT}/2)\\
&+\Big(\frac{1}{E^0_{-\downarrow}-E^0_{xy\uparrow}}+\frac{1}{E^0_{xy\downarrow}-E^0_{-\uparrow}}\Big)4\sin^2(\theta_{\rm JT}/2)+\frac{1}{E^0_{zx\downarrow}-E^0_{yz\uparrow}}+\frac{1}{E^0_{yz\downarrow}-E^0_{zx\uparrow}}\Big]\end{aligned}\tag{S41}$$

For the d^9 spin configuration, the second order correction to the energy can be obtained simply.

$$\begin{aligned}\Delta E^2_{d^9} = &\sum_{n=\text{occupied}}\Delta E^2_n = -\Delta E^2_{+\downarrow}\\
= &-\left(\tfrac{\zeta}{2}\right)^2\Big[\frac{(\cos(\theta_{\rm JT}/2)+\sqrt{3}\sin(\theta_{\rm JT}/2))^2(\cos^2\theta\cos^2\phi+\sin^2\phi)}{E^0_{+\downarrow}-E^0_{yz\uparrow}}\\
&+\frac{(\cos(\theta_{\rm JT}/2)-\sqrt{3}\sin(\theta_{\rm JT}/2))^2(\cos^2\theta\sin^2\phi+\cos^2\phi)}{E^0_{+\downarrow}-E^0_{zx\uparrow}}+\frac{4\cos^2(\theta_{\rm JT}/2)\sin^2\theta}{E^0_{+\downarrow}-E^0_{xy\uparrow}}\\
&+\frac{(\cos(\theta_{\rm JT}/2)+\sqrt{3}\sin(\theta_{\rm JT}/2))^2(\sin^2\theta\cos^2\phi)}{E^0_{+\downarrow}-E^0_{yz\downarrow}}\\
&+\frac{(\cos(\theta_{\rm JT}/2)-\sqrt{3}\sin(\theta_{\rm JT}/2))^2(\sin^2\theta\sin^2\phi)}{E^0_{+\downarrow}-E^0_{zx\downarrow}}+\frac{4\cos^2(\theta_{\rm JT}/2)\cos^2\theta}{E^0_{+\downarrow}-E^0_{xy\downarrow}}\Big]\end{aligned}\tag{S42}$$

The spin direction dependent part is

$$
\begin{aligned}
\Delta E^2_{d^9}(\mathbf{S}) = &\left(\tfrac{\zeta}{2}\right)^2 (\hat{\mathbf{s}}\cdot\mathbf{x})^2\Big[\Big(\frac{-1}{E^0_{+\downarrow}-E^0_{yz\downarrow}}+\frac{1}{E^0_{+\downarrow}-E^0_{yz\uparrow}}\Big)(\cos(\theta_{\mathrm{JT}}/2)+\sqrt{3}\sin(\theta_{\mathrm{JT}}/2))^2\Big] \\
&+\left(\tfrac{\zeta}{2}\right)^2 (\hat{\mathbf{s}}\cdot\mathbf{y})^2\Big[\Big(\frac{-1}{E^0_{+\downarrow}-E^0_{zx\downarrow}}+\frac{1}{E^0_{+\downarrow}-E^0_{zx\uparrow}}\Big)(\cos(\theta_{\mathrm{JT}}/2)-\sqrt{3}\sin(\theta_{\mathrm{JT}}/2))^2\Big] \\
&+\left(\tfrac{\zeta}{2}\right)^2 (\hat{\mathbf{s}}\cdot\mathbf{z})^2\Big[\Big(\frac{-1}{E^0_{+\downarrow}-E^0_{xy\downarrow}}+\frac{1}{E^0_{+\downarrow}-E^0_{xy\uparrow}}\Big)4\cos^2(\theta_{\mathrm{JT}}/2)\Big]
\end{aligned}
\tag{S43}
$$

Terms in the spin direction-dependent part of the second order energy corrections are divided into three parts corresponding to the local coordinate components, (x, y, z). Each directional part is again divided according to the dependency on the JT phase. Orbital ordering following the JT distortion affects the energy correction in two ways. One is the JT transformation of the e_g orbitals, which is explicitly expressed by the JT phase in Eq. (S41) and Eq. (S43). The other is the changes in the SOC-unperturbed orbital energies E^0_i which is implicit in the expressions Eq. (S41) and Eq. (S43). Because the crystal field splitting is larger than the changes by the JT effect, we can consider that the factors in the trigonometric functions of the JT phase in the Eq. (S41) and Eq. (S43) are leading factors to determine the MSIA direction. We can ignore the JT dependency of E^0_i's for simplicity. Note that the difference between the d^4 (Eq. (S41)) and d^9 (Eq. (S43)) cases rationalizes the difference in the favored axis of the spin between Cr- and **1-Cu**, although a comparison by evaluating all those terms is too complicated.

In $\Delta E^2_{d^4}(\mathbf{S})$, each directional part is composed of three sub-terms. Two of them are JT phase dependent and one is JT phase independent. Because we assumed $E^0_{e_g\uparrow} - E^0_{t_{2g}\uparrow} < E^0_{e_g\downarrow} - E^0_{t_{2g}\uparrow}$ and $E^0_{e_g\downarrow} - E^0_{t_{2g}\uparrow} > E^0_{t_{2g}\downarrow} - E^0_{e_g\uparrow} > 0$, we can determine whether each of the JT phase dependent sub-part is the energy-lowering or -raising term according to the sign of these energy-related factors. They are classified in Table. S5. JT phase independent terms are always energy-raising terms. Due to the phase difference by π of the JT phase dependent factors, the energy-lowering and -raising terms result in the same tendencies of the favored spin direction at a given θ_{JT}. On the other hand, $\Delta E^2_{d^9}(\mathbf{S})$ has only the energy-lowering

terms.

Table S5: Classification of the JT phase dependent according to the energy lowering and raising. $\Delta E < (>)\ 0$ means that the factor in energies multiplied to the listed JT phase dependent factor is negative (positive).

	$\Delta E < 0$	$\Delta E > 0$
$(\hat{\mathbf{s}} \cdot \mathbf{x})^2$	$(\cos(\theta_{\mathrm{JT}}/2) + \sqrt{3}\sin(\theta_{\mathrm{JT}}/2))^2$	$(\sin(\theta_{\mathrm{JT}}/2) - \sqrt{3}\cos(\theta_{\mathrm{JT}}/2))^2$
$(\hat{\mathbf{s}} \cdot \mathbf{y})^2$	$(\cos(\theta_{\mathrm{JT}}/2) - \sqrt{3}\sin(\theta_{\mathrm{JT}}/2))^2$	$(\sin(\theta_{\mathrm{JT}}/2) + \sqrt{3}\cos(\theta_{\mathrm{JT}}/2))^2$
$(\hat{\mathbf{s}} \cdot \mathbf{z})^2$	$4\cos^2(\theta_{\mathrm{JT}}/2)$	$4\sin^2(\theta_{\mathrm{JT}}/2)$

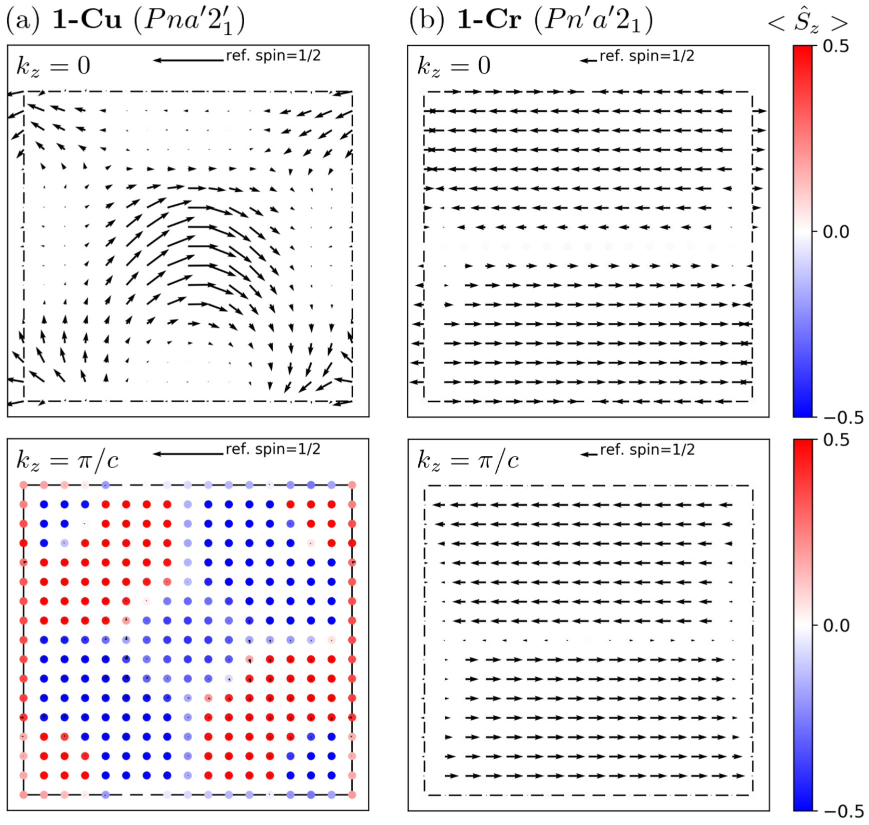

Figure S11: The spin textures calculated by using OpenMX. (a) **1-Cu** and (b) **1-Cr**, at the $k_z = 0$ (upper panels) and $k_z = \pi/c$ (lower panels) planes. The in-plane x and y components are represented by arrows of which the length is the in-plane magnitude with respect to the reference spin 1/2 above the figure. The z components are represented by colormap of dots. The inner boundary of each figure coincides with the Brillouin zone boundary.

S11. Spin and Orbital Textures in k-space

The spin texture in the k-space of the **1-Cr/Cu** shows interesting features. The spin texture is defined as $\mathbf{s}_{\mathbf{k}n} = \langle \psi_{\mathbf{k}n} | \mathbf{S} | \psi_{\mathbf{k}n} \rangle$ for a specific band n. The spin texture of the highest occupied band of each compound at $k_z = 0$ and $k_z = \pi/c$ planes are shown in Fig. 7 in the main text. For Fig. 7, the spin expectation values are extracted from the projected values to each atomic state, which are stored in the output file PROCAR of VASP.

Since we observed that the orbital magnetic moment is a dominant contribution to the magnetic moment of the **1-Cu** and a local orbital magnetic moment is not parallel to the local spin moment in both **1-Cr/Cu**s, it is interesting to investigate the orbital texture.

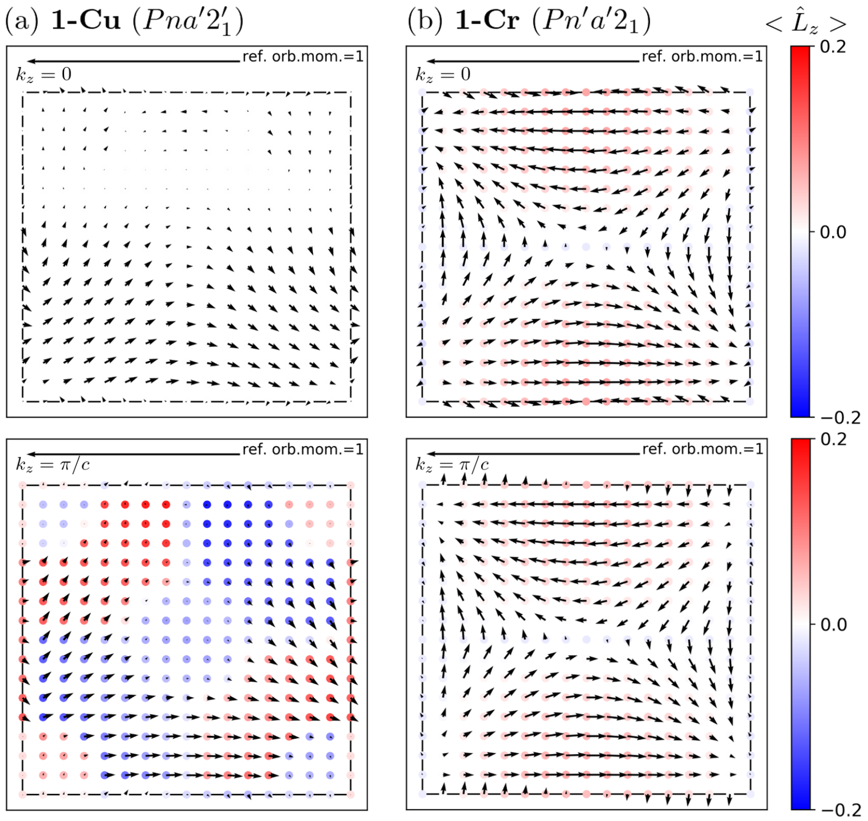

Figure S12: The orbital angular momentum textures calculated by using OpenMX. (a) **1-Cu** and (b) **1-Cr**, at the $k_z = 0$ (upper panels) and $k_z = \pi/c$ (lower panels) planes. The in-plane x and y components are represented by arrows of which the length is the in-plane magnitude with respect to the reference orbital angular momentum 1 above the figure. The z components are represented by colormap of dots. The inner boundary of each figure coincides with the Brillouin zone boundary.

In our context, the orbital texture is the orbital angular momentum texture defined as $\mathbf{l}_{\mathbf{k}n} = \langle \psi_{\mathbf{k}n} | \mathbf{L} | \psi_{\mathbf{k}n} \rangle$. For this purpose, we adopted another first-principles calculation package OpenMX,[S12] which adopts the linear combination of the pseudo atomic orbital (LCPAO) method. Therefore, orbital angular momentum can be calculated at an atomic orbital angular momentum operator level. As a LCPAO basis, we adopted `H6.0-s2p2`, `C6.0-s2p2`, `N6.0-s2p2`, `O6.0-s2p2d1`, `Cu6.0H-s2p2d2f1`, and `Cr6.0-s2p2d2f1`, which indicate the number of the set of each s, p, d, and f orbital sets for each atomic species. Other computational settings are compatible with the setting for the VASP package.

As a preliminary step, we calculated the spin texture using OpenMX[S13] to check if the

two software packages give compatible results. As shown in Fig. S11, the main features of the spin textures are consistent: In the **1-Cu**, the curly in-plane texture at $k_z = 0$ and the alignment to z-direction at $k_z = \pi/c$ (Fig. S11 (a)); In the **1-Cr**, the persistent type spin textures (Fig. S11 (b)). On the other hand, there are some differences in the case of **1-Cu**. The magnitude of the spins are larger in the OpenMX data at $k_z = 0$, and the signs of the z components at $k_z = \pi/c$ show a different pattern. Nevertheless, we can expect that primary features will be shared in the OpenMX results.

The orbital textures calculated by OpenMX are shown in Fig. S12. The orbital angular momenta are calculated using an in-house post-processing program for OpenMX. Interestingly, the orbital textures are not parallel to the spin textures at all k-points. In the **1-Cu** (Fig. S12 (a)), there are no out-of-plane components at $k_z = 0$ plane, and their in-plane components show a different pattern from the spin texture. At $k_z = \pi/c$, the out-of-plane orbital texture shows a similar texture with the spin besides their magnitude. However, there are also in-plane components in the orbital texture. In the **1-Cr** (Fig. S12 (b)), interestingly, the in-plane orbital texture shows a Dresselhaus-type texture[S14] at both $k_z = 0$ and $k_z = \pi/c$ planes, i.e., the coexistence of the persistent spin texture and the Dresselhaus orbital texture. Also, there are small out-of-plane components in the orbital texture.

References

[S1] Diffraction, R. O. CrysAliPro Software system. 2018.

[S2] Sheldrick, G. M. SHELXT - Integrated space-group and crystal-structure determination. *Acta Cryst A* **2015**, *71*, 3–8.

[S3] Dolomanov, O. V.; Bourhis, L. J.; Gildea, R. J.; Howard, J. a. K.; Puschmann, H. OLEX2: a complete structure solution, refinement and analysis program. *J Appl Cryst* **2009**, *42*, 339–341.

[S4] Sheldrick, G. M. Crystal structure refinement with SHELXL. *Acta Cryst C* **2015**, *71*, 3–8.

[S5] Flor, G. d. l.; Orobengoa, D.; Tasci, E.; Perez-Mato, J. M.; Aroyo, M. I. Comparison of structures applying the tools available at the Bilbao Crystallographic Server. *J. Appl. Cryst.* **2016**, *49*, 653–664.

[S6] Stroppa, A.; Jain, P.; Barone, P.; Marsman, M.; Perez-Mato, J. M.; Cheetham, A. K.; Kroto, H. W.; Picozzi, S. Electric Control of Magnetization and Interplay between Orbital Ordering and Ferroelectricity in a Multiferroic Metal-Organic Framework. *Angew. Chem. Int. Ed.* **2011**, *50*, 5847–5850.

[S7] Stroppa, A.; Barone, P.; Jain, P.; Perez-Mato, J. M.; Picozzi, S. Hybrid Improper Ferroelectricity in a Multiferroic and Magnetoelectric Metal-Organic Framework. *Adv. Mater.* **2013**, *25*, 2284–2290.

[S8] Dzyaloshinsky, I. A thermodynamic theory of "weak" ferromagnetism of antiferromagnetics. *J. Phys. Chem. Solids* **1958**, *4*, 241–255.

[S9] Moriya, T. Anisotropic Superexchange Interaction and Weak Ferromagnetism. *Phys. Rev.* **1960**, *120*, 91–98.

[S10] Takayama, H.; Bohnen, K.-P.; Fulde, P. Magnetic surface anisotropy of transition metals. *Phys. Rev. B* **1976**, *14*, 2287–2295.

[S11] Griffith, J. S. *The theory of transition-metal ions*; Cambridge University Press, 1971.

[S12] Ozaki, T. Variationally optimized atomic orbitals for large-scale electronic structures. *Phys. Rev. B* **2003**, *67*, 155108, website: openmx-square.org.

[S13] Kotaka, H.; Ishii, F.; Saito, M. Rashba Effect on the Structure of the Bi One-Bilayer Film: Fully Relativistic First-Principles Calculation. *Jpn. J. Appl. Phys.* **2013**, *52*, 035204.

[S14] Winkler, R. *Spin-Orbit Coupling Effects in Two-Dimensional Electron and Hole Systems*; Springer Tracts in Modern Physics; Springer, pp 69–129.
